# Associations of the *APOB* rs693 and rs17240441 polymorphisms with plasma APOB and lipid levels: a meta-analysis

**DOI:** 10.1186/s12944-017-0558-7

**Published:** 2017-09-06

**Authors:** Caiqin Niu, Zhi Luo, Liuqin Yu, Yang Yang, Yun Chen, Xin Luo, Feiya Lai, Yongyan Song

**Affiliations:** 1 Department of Traditional Chinese Medicine, Affiliated Hospital of North Sichuan Medical College, Nanchong, 637000 People’s Republic of China; 2 Department of Cardiology, Affiliated Hospital of North Sichuan Medical College, Nanchong, 637000 People’s Republic of China; 3 Institute of Materia Medica, School of Pharmacy, North Sichuan Medical College, Nanchong, 637000 People’s Republic of China; 4 School of Clinical Medicine, North Sichuan Medical College, Nanchong, 637000 People’s Republic of China; 5 Department of Medical Biochemistry, School of Preclinical Medicine, North Sichuan Medical College, Nanchong, 637000 People’s Republic of China

**Keywords:** *APOB*, rs693, rs17240441, Polymorphism, Lipid

## Abstract

**Background:**

The associations of the apolipoprotein B gene (*APOB*) rs693 and rs17240441 polymorphisms with plasma levels of APOB and lipids have been widely explored, but the results were inconclusive. This meta-analysis aimed to clarify the associations of the rs693 and rs17240441 polymorphisms with fasting APOB and lipid levels.

**Methods:**

Sixty-one studies (50,018 subjects) and 23 studies (8425 subjects) were respectively identified for the rs693 and rs17240441 polymorphisms by searching in PubMed, Google Scholar, Web of Science, Cochrane Library, Wanfang, VIP and CNKI databases. The following information was collected for each study: first author, age, gender, ethnicity, health condition, sample size, genotyping, lipid assay method, mean and standard deviation or standard error of APOB and lipid variables by genotypes. A dominant model was used for this meta-analysis.

**Results:**

The carriers of the rs693 variant allele (T) had higher levels of APOB [standardized mean difference (SMD) = 0.26, 95% confidence interval (CI) = 0.16–0.36, *P* < 0.01], triglycerides (TG) (SMD = 0.12, 95% CI = 0.05–0.20, *P* < 0.01), total cholesterol (TC) (SMD = 0.24, 95% CI = 0.17–0.30, *P* < 0.01) and low-density lipoprotein cholesterol (LDL-C) (SMD = 0.22, 95% CI = 0.14–0.30, *P* < 0.01), and lower levels of high-density lipoprotein cholesterol (HDL-C) (SMD = −0.06, 95% CI = −0.11–0.01, *P* = 0.01) than the non-carriers. The carriers of the rs17240441 deletion allele had higher levels of APOB (SMD = 0.13, 95% CI = 0.06–0.20, *P* < 0.01), TC (SMD = 0.17, 95% CI = 0.07–0.26, *P* < 0.01) and LDL-C (SMD = 0.15, 95% CI = 0.07–0.23, *P* < 0.01) than the non-carriers.

**Conclusions:**

The rs693 polymorphism is significantly associated with higher levels of APOB, TG, TC and LDL-C, and lower levels of HDL-C. The rs17240441 polymorphism is significantly associated with higher levels of APOB, TC and LDL-C. Further studies are needed to elucidate the underlying mechanisms.

**Electronic supplementary material:**

The online version of this article (10.1186/s12944-017-0558-7) contains supplementary material, which is available to authorized users.

## Background

Coronary heart disease (CHD) is currently a leading cause of death in developed countries, and in some developing countries including China [1]. CHD is a multifactorial disease and a number of CHD risk factors have been identified in the past few decades. Dyslipidaemia is one of the most important risk factors for CHD and accounts for at least 50% of the population-attributable risk [2]. Dyslipidaemia is characterized by increased levels of apolipoprotein (APO) B, triglycerides (TG), total cholesterol (TC) and low-density lipoprotein cholesterol (LDL-C), and/or decreased levels of APOAI and high-density lipoprotein cholesterol (HDL-C) in circulation. Over the last few decades, intensive efforts have been made in the scientific community to investigate the associations between the polymorphisms in apolipoprotein genes and plasma lipid levels, but the results were not consistent across the studies. It is difficult to identify the dyslipidaemia-related genetic polymorphisms successfully due to various reasons such as small sample size.

APOB plays an important role in lipoprotein metabolism. In the circulation, each particle of the atherogenic lipoproteins [i.e. chylomicron, very low-density lipoprotein (VLDL), intermediary density lipoprotein (IDL), low-density lipoprotein (LDL) and lipoprotein (a)] carries one APOB molecule, so high level of APOB is directly associated with high levels of lipids including TG, TC and/or LDL-C. APOB is divided into APOB100 and APOB48 according to their molecular sizes. APOB100 and APOB48 are encoded by the same *APOB* gene. The *APOB* gene contains 29 exons and 28 introns with a total length of 43 kb, and is located on the short arm of human chromosome 2 (p23–24) [3]. The *APOB* gene is highly polymorphic, and there are over 5000 polymorphic sites in or around the *APOB* gene (https://www.ncbi.nlm.nih.gov/snp/?term=*APOB*). These polymorphic loci can be divided into single nucleotide polymorphisms (SNPs), insertion/deletion polymorphisms, and small tandem repeat polymorphisms according to the characteristics of nucleotide sequences. Two SNPs (rs693 and rs17240441) within the *APOB* gene have been extensively studied in respect of their associations with plasma lipid levels and CHD risk over the past three decades. The rs693 polymorphism is located in exon 26 of the *APOB* gene and formed by a transition from C to T [4]. The 2488th genetic code of the *APOB* gene is accordingly changed from ACC to ACT. However, the rs693 polymorphism is a synonymous mutation and the amino acid residue (Thr) is not changed after the nucleotide replacement. The minor allele T was widely reported to be a risk allele for CHD [5] and its frequency is 0.02–0.10 in Asians, 0.49–0.50 in Caucasians, and 0.15–0.23 in Africans. The rs17240441 polymorphism is located in the first exon of the *APOB* gene [6]. It is formed by the insertion/deletion of a nine-nucleotide sequence (GCAGCGCCA) in exon 1, resulting in insertion/deletion of 3 amino acid residues (Arg-Glu-Val) in the signal peptide of APOB. The insertion allele (I) contains a complete signal peptide of 27 amino acid residues, whereas the deletion allele (D) only contains 24 amino acid residues. Research results showed that the D allele was a risk allele for CHD [6, 7] and the frequency of the D allele is 0.12–0.39 in Asians, 0.21–0.64 in Caucasians, and 0.23–0.68 in Africans.

Although there were a large number of studies investigating the associations of the two polymorphic loci with plasma APOB and lipid levels, the results were inconsistent and inconclusive. In some of these studies, the rs693 polymorphism was reported to be associated with higher levels of APOB [8–18], TG [19–22], TC [23–30] and LDL-C [30–32], and lower levels of HDL-C [33–37]; the rs17240441 polymorphism was associated with higher levels of APOB [38–40], TG [41–43], TC [44–46], LDL-C [44–46], and lower levels of HDL-C [36, 42]. However, the results obtained from other studies did not support these findings [47–56]. Hence, a meta-analysis is required to clarify the relationships between the two polymorphisms and plasma levels of APOB and lipids.

In this paper, a meta-analysis was performed based on previous publications to investigate the associations of the rs693 and rs17240441 polymorphisms with fasting APOB and lipid levels. Our analysis results can provide the opportunity to unveil the interrelationships among the rs693 and rs17240441 polymorphisms, dyslipidaemia and CHD.

## Results

### Characteristics of the included studies

Initial search of the databases yielded 1364 articles. One thousand one hundred and seventy-nine articles were excluded according to titles and abstracts. Then full-text articles were retrieved and assessed on the basis of the inclusion criteria. One hundred and nine articles were ineligible for the following reasons: 30 articles did not provide complete data for this meta-analysis, 68 articles presented data for other polymorphisms, 8 articles had subjects overlapping with other publications, and 3 articles were based on pedigree data. In the end, 76 studies [4, 6, 8–81] were selected for this meta-analysis.

The characteristics of the 76 included studies are summarized in Additional file 1: Table S1. Sixty-one studies [4, 8–38, 48–54, 57, 58, 60, 61, 63–80] presented the APOB and/or lipid levels for the rs693 polymorphism by genotypes. Among them, 39 studies [8–18, 24, 28, 30, 31, 33, 35, 36, 38, 48–56, 58, 64, 67, 69, 70, 75–80], 53 studies [4, 8–16, 18–24, 26, 28, 30–33, 35, 36, 38, 48–58, 60, 63, 65–68, 70–73, 75–80], 60 studies [4, 8–21, 23–36, 38, 48–57, 60, 61, 63–80], 52 studies [4, 8–16, 19–26, 28, 30–33, 35, 36, 38, 48–57, 60, 64–66, 68–71, 73–80] and 48 studies [4, 8–16, 20, 21, 24, 26, 28–37, 48–57, 63–66, 68, 70, 73–80] presented the data for APOB, TG, TC, LDL-C and HDL-C, respectively. Twenty-three studies [6, 21, 25, 26, 34, 36, 38–47, 52, 57, 59, 61, 62, 65, 81] presented the APOB and/or lipid levels for the rs17240441 polymorphism by genotypes, and 12 studies [6, 36, 38–40, 43–47, 52, 81], 19 studies [6, 21, 26, 36, 38, 39, 41–47, 52, 57, 59, 62, 65, 81], 22 studies [6, 21, 25, 26, 34, 36, 38–47, 52, 57, 61, 62, 65, 81], 20 studies [6, 21, 25, 26, 36, 38, 40–47, 52, 57, 59, 62, 65, 81] and 18 studies [6, 21, 26, 34, 36, 40–47, 52, 57, 62, 65, 81] of which presented the data for APOB, TG, TC, LDL-C and HDL-C, respectively. Thirty-two studies [9, 11, 14, 17–20, 23–25, 27–29, 32, 33, 37–39, 42, 43, 45–50, 54, 59, 61–63, 69], 35 studies [8, 12, 13, 15, 16, 21, 22, 30, 31, 34–36, 40, 41, 44, 51–53, 55, 56, 58, 60, 64, 68, 71–81] and 8 studies [4, 6, 10, 26, 57, 65–67] involved Caucasians, Asians, and the subjects of other ethnic origins, respectively. Twelve studies [16, 17, 23, 24, 32–34, 37, 39, 50, 58, 63] and 1 study [27] respectively involved males and females, and the rest 52 studies [4, 6, 8–15, 18–22, 25, 26, 28–31, 35, 36, 38, 51–57, 59–62, 64–69, 71–81] involved both males and females, among which 11 studies [10, 11, 21, 25, 41, 48, 49, 51, 56, 61, 62] separately provided data for males and females. Twenty-one studies [4, 6, 9, 11, 16, 23, 27, 33, 35–37, 39, 41, 45, 47, 50, 53, 61, 63, 65, 66], 8 studies [18, 32, 54, 59, 72, 74, 76, 80], 2 studies [46, 68], 3 studies [20, 67, 69], 3 studies [30, 71, 73], 4 studies [12, 55, 77, 79], 1 study [78] and 1 study [26] involved CHD, dyslipidaemia, overweight or obese, diabetes, cerebral infarction or hemorrhage, gallstone, nephropathy and HIV-1 patients, respectively. Thirty-nine studies [4, 8, 10–12, 21, 23, 25, 27, 29, 30, 33, 35–37, 41, 45, 47–49, 51, 53–56, 59, 61–63, 65–68, 71, 72, 76, 77, 79, 80] separately presented data for more than one subpopulation, and each subpopulation was treated as a separate comparison.

### Summary statistics

One hundred and two comparisons and 34 comparisons were respectively distinguished for the rs693 and rs17240441 polymorphisms according to the categories such as gender, ethnicity and health condition. Sixty-three, 85, 98, 82 and 81 comparisons were respectively included to compare the differences in APOB, TG, TC, LDL-C and HDL-C for the rs693 polymorphism (Table 1). Fifteen, 29, 32, 30 and 27 comparisons were respectively included to compare the differences in APOB, TG, TC, LDL-C and HDL-C for the rs17240441 polymorphism (Table 2).

**Table 1:** Meta-analysis of the *APOB* rs693 polymorphism with plasma APOB and lipid levels

Groups or subgroups	Comparisons (Subjects)	*P*_Heterogeneity_	SMD (95% CI)	*P*_SMD_
APOB				
All	63 (12,364)	< 0.01	0.26 (0.16–0.36)	< 0.01
Studies in HWE	53 (10,818)	< 0.01	0.25 (0.14–0.36)	< 0.01
Male	14 (2620)	0.13	0.12 (0.01–0.24)	0.03
Female	7 (1014)	0.37	0.29 (0.12–0.45)	< 0.01
Caucasian	21 (5512)	< 0.01	0.19 (0.08–0.30)	< 0.01
Asian	38 (6129)	< 0.01	0.34 (0.16–0.51)	< 0.01
CHD	9 (1198)	0.03	0.17 (−0.05–0.38)	0.12
Healthy or control	39 (8829)	< 0.01	0.25 (0.12–0.38)	< 0.01
Case-control studies	34 (4971)	< 0.01	0.15 (0.05–0.25)	< 0.01
Cohort studies	29 (7393)	< 0.01	0.41 (0.24–0.57)	< 0.01
TG				
All	85 (22,128)	< 0.01	0.12 (0.05–0.20)	< 0.01
Studies in HWE	71 (19,590)	< 0.01	0.13 (0.05–0.21)	< 0.01
Male	19 (2867)	0.05	0.00 (−0.12–0.11)	0.95
Female	8 (1077)	0.65	0.10 (−0.05–0.25)	0.19
Caucasian	26 (5454)	0.14	0.02 (−0.06–0.10)	0.65
Asian	47 (14,257)	< 0.01	0.25 (0.12–0.37)	< 0.01
CHD	14 (1716)	< 0.01	0.11 (−0.09–0.30)	0.28
Healthy or control	50 (17,429)	< 0.01	0.09 (0.00–0.18)	0.04
Case-control studies	49 (6571)	< 0.01	0.11 (0.00–0.23)	0.05
Cohort studies	36 (15,557)	< 0.01	0.13 (0.03–0.22)	0.01
TC				
All	98 (41,764)	< 0.01	0.24 (0.17–0.30)	< 0.01
Studies in HWE	80 (30,373)	< 0.01	0.26 (0.18–0.34)	< 0.01
Male	22 (3594)	0.18	0.14 (0.05–0.22)	< 0.01
Female	12 (1362)	< 0.01	0.14 (−0.09–0.37)	0.22
Caucasian	38 (32,268)	< 0.01	0.14 (0.09–0.20)	< 0.01
Asian	48 (7080)	< 0.01	0.45 (0.25–0.66)	< 0.01
CHD	17 (2076)	0.34	0.13 (0.03–0.24)	0.01
Healthy or control	52 (10,745)	< 0.01	0.28 (0.14–0.41)	< 0.01
Case-control studies	51 (6734)	< 0.01	0.19 (0.10–0.28)	< 0.01
Cohort studies	47 (35,030)	< 0.01	0.29 (0.19–0.38)	< 0.01
LDL-C				
All	82 (22,286)	< 0.01	0.22 (0.14–0.30)	< 0.01
Studies in HWE	67 (19,724)	< 0.01	0.25 (0.16–0.34)	< 0.01
Male	18 (2685)	0.83	0.18 (0.10–0.27)	< 0.01
Female	9 (1138)	< 0.01	0.05 (−0.23–0.33)	0.72
Caucasian	26 (5893)	0.04	0.17 (0.08–0.26)	< 0.01
Asian	46 (14,268)	< 0.01	0.33 (0.19–0.48)	< 0.01
CHD	13 (1624)	0.03	0.07 (−0.09–0.24)	0.38
Healthy or control	48 (17,243)	< 0.01	0.23 (0.13–0.33)	< 0.01
Case-control studies	42 (5871)	< 0.01	0.14 (0.05–0.24)	< 0.01
Cohort studies	40 (16,415)	< 0.01	0.31 (0.19–0.44)	< 0.01
HDL-C				
All	81 (39,292)	< 0.01	−0.06 (−0.11–0.01)	0.01
Studies in HWE	66 (28,316)	< 0.01	−0.04 (−0.10–0.01)	0.12
Male	19 (2912)	0.03	−0.08 (−0.19–0.03)	0.17
Female	8 (1079)	0.54	−0.02 (−0.17–0.14)	0.84
Caucasian	26 (30,367)	0.05	−0.04 (−0.08–0.01)	0.11
Asian	45 (6748)	< 0.01	−0.08 (−0.19–0.02)	0.12
CHD	13 (1665)	0.02	−0.15 (−0.32–0.01)	0.07
Healthy or control	46 (9700)	< 0.01	−0.03 (−0.11–0.05)	0.49
Case-control studies	43 (6020)	< 0.01	−0.05 (−0.14–0.04)	0.29
Cohort studies	38 (33,272)	< 0.01	−0.06 (−0.11–0.00)	0.04

*SMD* standardized mean difference, *95% CI* 95% confidence interval, *HWE* Hardy-Weinberg equilibrium, *APOB* apolipoprotein B, *TG* triglyceride, *TC* total cholesterol, *LDL-C* low-density lipoprotein cholesterol, *HDL-C* high-density lipoprotein cholesterol

**Table 2:** Meta-analysis of the *APOB* rs17240441 polymorphism with plasma APOB and lipid levels

Groups or subgroups	Comparisons (Subjects)	*P*_Heterogeneity_	SMD (95% CI)	*P*_SMD_
APOB				
All	15 (5047)	0.27	0.13 (0.06–0.20)	< 0.01
Studies in HWE	12 (4192)	0.32	0.11 (0.04–0.19)	< 0.01
Caucasian	8 (3626)	0.31	0.12 (0.03–0.21)	< 0.01
Asian	6 (1264)	0.18	0.14 (−0.01–0.30)	0.07
CHD	5 (2841)	0.86	0.11 (0.03–0.18)	< 0.01
Healthy or control	9 (1975)	0.06	0.12 (−0.03–0.26)	0.11
Case-control studies	8 (3951)	0.29	0.13 (0.04–0.21)	< 0.01
Cohort studies	7 (1096)	0.23	0.15 (−0.001–0.30)	0.05
TG				
All	29 (7411)	< 0.01	0.03 (−0.04–0.11)	0.37
Studies in HWE	24 (6576)	0.02	0.04 (−0.05–0.12)	0.44
Male	6 (3215)	0.14	0.11 (−0.01–0.24)	0.08
Female	4 (411)	0.16	0.22 (−0.10–0.53)	0.18
Caucasian	12 (4691)	0.11	0.02 (−0.11–0.07)	0.62
Asian	11 (1281)	0.13	0.21 (0.06–0.36)	< 0.01
CHD	8 (3138)	0.02	0.15 (−0.01–0.32)	0.07
Healthy or control	18 (3274)	0.04	−0.02 (−0.13–0.08)	0.70
Case-control studies	15 (4538)	0.12	0.09 (−0.003–0.18)	0.06
Cohort studies	14 (2873)	0.03	0.03 (−0.04–0.11)	0.60
TC				
All	32 (7875)	< 0.01	0.17 (0.07–0.26)	< 0.01
Studies in HWE	23 (6133)	< 0.01	0.18 (0.04–0.33)	0.01
Male	7 (3220)	0.72	0.14 (0.07–0.21)	< 0.01
Female	5 (470)	< 0.01	0.74 (−0.26–1.75)	0.15
Caucasian	14 (4598)	0.34	0.13 (0.06–0.20)	< 0.01
Asian	13 (1924)	< 0.01	0.33 (0.06–0.60)	0.02
CHD	10 (3455)	0.42	0.12 (0.05–0.19)	< 0.01
Healthy or control	15 (2577)	< 0.01	0.23 (0.05–0.40)	0.01
Case-control studies	14 (4526)	< 0.01	0.22 (0.03–0.41)	0.02
Cohort studies	18 (3349)	0.15	0.14 (0.05–0.23)	< 0.01
LDL-C				
All	30 (5658)	< 0.01	0.15 (0.07–0.23)	< 0.01
Studies in HWE	23 (4233)	< 0.01	0.15 (0.04–0.25)	< 0.01
Male	5 (955)	0.85	0.07 (−0.06–0.21)	0.27
Female	4 (411)	0.04	0.01 (−0.38–0.41)	0.95
Caucasian	13 (2562)	0.38	0.14 (0.05–0.23)	< 0.01
Asian	12 (1817)	< 0.01	0.18 (0.00–0.35)	0.05
CHD	7 (879)	0.09	0.15 (−0.04–0.34)	0.12
Healthy or control	19 (3764)	< 0.01	0.13 (0.02–0.24)	0.02
Case-control studies	15 (2807)	0.07	0.12 (0.02–0.23)	0.03
Cohort studies	15 (2851)	< 0.01	0.18 (0.06–0.31)	< 0.01
HDL-C				
All	27 (5124)	< 0.01	−0.04 (−0.12–0.05)	0.40
Studies in HWE	21 (3750)	< 0.01	−0.07 (−0.18–0.05)	0.25
Male	5 (703)	0.10	−0.06 (−0.30–0.17)	0.58
Female	4 (411)	0.57	−0.01 (−0.23–0.21)	0.91
Caucasian	9 (1881)	0.30	−0.02 (−0.13–0.09)	0.77
Asian	13 (1924)	< 0.01	−0.10 (−0.26–0.06)	0.21
CHD	7 (879)	0.05	−0.06 (−0.27–0.14)	0.55
Healthy or control	18 (3605)	0.01	−0.03 (−0.13–0.08)	0.65
Case-control studies	13 (2267)	0.02	−0.07 (−0.20–0.06)	0.31
Cohort studies	14 (2854)	0.06	−0.01 (−0.12–0.10)	0.89

*SMD* standardized mean difference, *95% CI* 95% confidence interval, *HWE* Hardy-Weinberg equilibrium, *APOB* apolipoprotein B, *TG* triglyceride, *TC* total cholesterol, *LDL-C* low-density lipoprotein cholesterol, *HDL-C* high-density lipoprotein cholesterol

Fifty thousand and eighteen subjects and 8425 subjects were respectively enrolled in the analyses for the rs693 and rs17240441 polymorphisms. For the rs693 polymorphism, 45.0% of the subjects (22,503 subjects) had the CC genotype, and 55.0% of them (27,515 subjects) had the CT or TT genotype. For the rs17240441 polymorphism, 48.9% of the subjects (4117 subjects) had the II genotype, and 51.1% of them (4308 subjects) had the ID or DD genotype.

### Associations of the *APOB* rs693 polymorphism with APOB and lipid levels

**Fig. 1:**
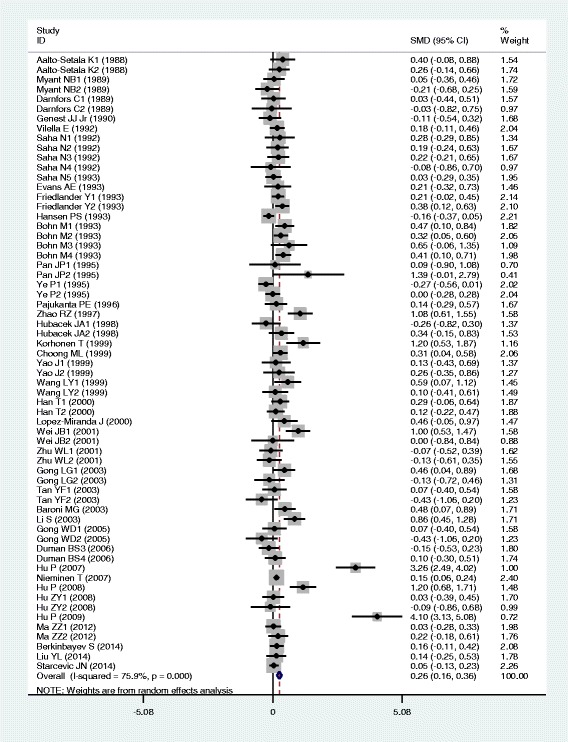
Forest plot of the meta-analysis between *APOB* rs693 polymorphism and plasma APOB levels (63 comparisons and 12,364 subjects were included)

**Fig. 2:**
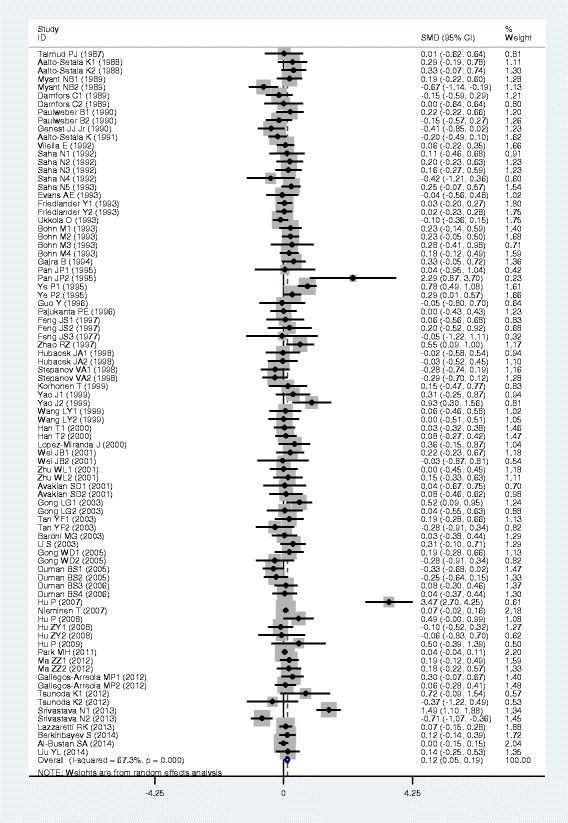
Forest plot of the meta-analysis between APOB rs693 polymorphism and plasma TG levels (85 comparisons 22,128 subjects were included)

**Fig. 3:**
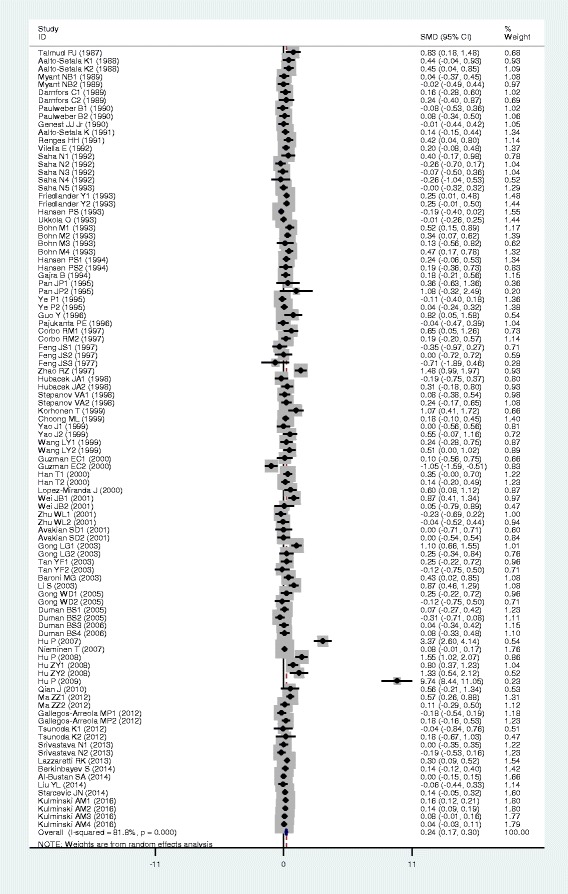
Forest plot of the meta-analysis between *APOB* rs693 polymorphism and plasma TC levels (98 comparisons and 41,764 subjects were included)

**Fig. 4:**
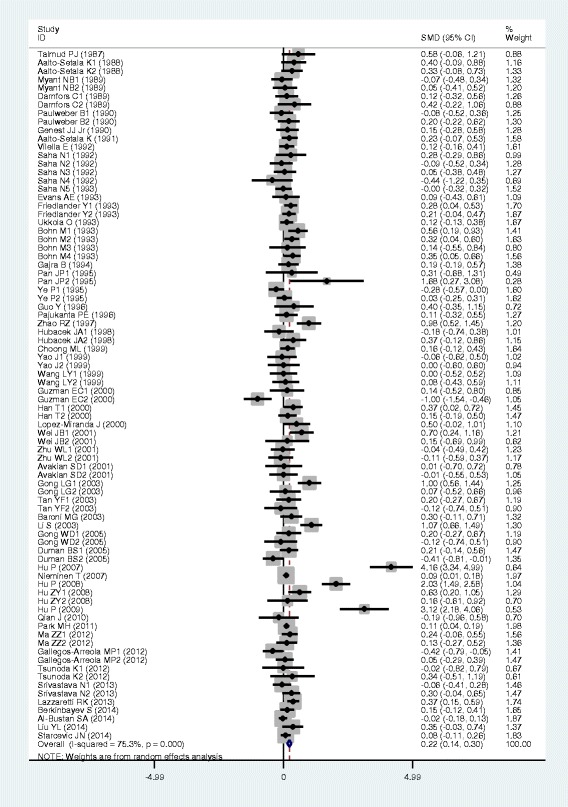
Forest plot of the meta-analysis between *APOB* rs693 polymorphism and plasma LDL-C levels (82 comparisons and 22,286 subjects were included)

**Fig. 5:**
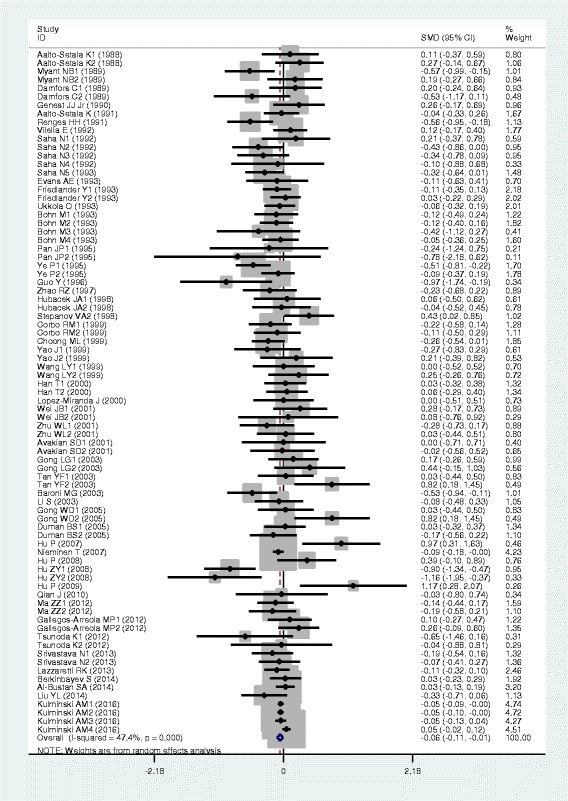
Forest plot of the meta-analysis between *APOB* rs693 polymorphism and plasma HDL-C levels (81 comparisons and 39,292 subjects were included)

The outcomes of the analyses on all comparisons for the rs693 polymorphism showed that the T carriers had higher levels of APOB [standardized mean difference (SMD) = 0.26, 95% confidence interval (CI) = 0.16–0.36, *P* < 0.01), TG (SMD = 0.12, 95% CI = 0.05–0.20, *P* < 0.01), TC (SMD = 0.24, 95% CI = 0.17–0.30, *P* < 0.01) and LDL-C (SMD = 0.22, 95% CI = 0.14–0.30, *P* < 0.01), and lower levels of HDL-C (SMD = −0.06, 95% CI = −0.11–0.01, *P* = 0.01) than the non-carriers (Table 1, Figs. 1, 2, 3, 4 and 5). When the analyses were limited to the studies in Hardy-Weinberg equilibrium (HWE), the significant associations between the rs693 polymorphism and higher levels of APOB (SMD = 0.25, 95% CI = 0.14–0.36, *P* < 0.01), TG (SMD = 0.13, 95% CI = 0.05–0.21, *P* < 0.01), TC (SMD = 0.26, 95% CI = 0.18–0.34, *P* < 0.01) and LDL-C (SMD = 0.25, 95% CI = 0.16–0.34, *P* < 0.01) were also detected (Table 1). In the cumulative analyses according to the publication years between the rs693 polymorphism and lipid levels, the associations became significant from years 1993, 1995, 1977, 1988 and 1993 for APOB, TG, TC, LDL-C and HDL-C, respectively (Additional file 1: Figures S20-S24).

Then the subgroup analyses stratified by the characteristics of subjects and the types of studies were performed. The significant association between the rs693 polymorphism and higher levels of APOB was detected in both males (SMD = 0.12, 95% CI = 0.01–0.24, *P* = 0.03) and females (SMD = 0.29, 95% CI = 0.12–0.45, *P* < 0.01), whereas the associations between the rs693 polymorphism and higher levels of TC (SMD = 0.14, 95% CI = 0.05–0.22, *P* < 0.01) and LDL-C (SMD = 0.18, 95% CI = 0.10–0.27, *P* < 0.01) were found to be significant only in males, but not in females. The significant associations between the rs693 polymorphism and higher levels of APOB (SMD = 0.34, 95% CI = 0.16–0.51, *P* < 0.01), TG (SMD = 0.25, 95% CI = 0.12–0.37, *P* < 0.01), TC (SMD = 0.45, 95% CI = 0.25–0.66, *P* < 0.01) and LDL-C (SMD = 0.33, 95% CI = 0.19–0.48, *P* < 0.01) were detected in Asians. In Caucasian subjects, the significant associations of the rs693 polymorphism with higher levels of APOB (SMD = 0.19, 95% CI = 0.08–0.30, *P* < 0.01), TC (SMD = 0.14, 95% CI = 0.09–0.20, *P* < 0.01) and LDL-C (SMD = 0.17, 95% CI = 0.08–0.26, *P* < 0.01) were detected. The significant association between the rs693 polymorphism and higher levels of TC was detected in both the CHD patients (SMD = 0.13, 95% CI = 0.03–0.24, *P* = 0.01) and the healthy/control subjects (SMD = 0.28, 95% CI = 0.14–0.41, *P* < 0.01). The significant associations between the rs693 polymorphism and higher levels of APOB (SMD = 0.25, 95% CI = 0.12–0.38, *P* < 0.01), TG (SMD = 0.09, 95% CI = 0.00–0.18, *P* = 0.04) and LDL-C (SMD = 0.23, 95% CI = 0.13–0.33, *P* < 0.01) were detected in the healthy/control subjects, but not in the patients with CHD. When the types of studies were taken into account, the significant associations between the rs693 polymorphism and higher levels of APOB (SMD = 0.41, 95% CI = 0.24–0.57, *P* < 0.01), TG (SMD = 0.13, 95% CI = 0.03–0.22, *P* = 0.01), TC (SMD = 0.29, 95% CI = 0.19–0.38, *P* < 0.01) and LDL-C (SMD = 0.31, 95% CI = 0.19–0.44, *P* < 0.01), and lower levels of HDL-C (SMD = −0.06, 95% CI = −0.11-0.00, *P* = 0.04) were detected in the cohort studies. The significant associations between the rs693 polymorphism and higher levels of APOB (SMD = 0.15, 95% CI = 0.05–0.25, *P* < 0.01), TG (SMD = 0.11, 95% CI = 0.00–0.23, *P* = 0.05), TC (SMD = 0.19, 95% CI = 0.10–0.28, *P* < 0.01) and LDL-C (SMD = 0.14, 95% CI = 0.05–0.24, *P* < 0.01) were detected in the case-control studies.

### Associations of the *APOB* rs17240441 polymorphism with APOB and lipid levels

**Fig. 6:**
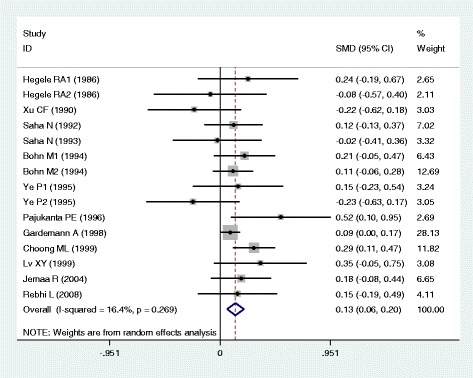
Forest plot of the meta-analysis between *APOB* rs17240441 polymorphism and plasma APOB levels (15 comparisons and 5047 subjects were included)

**Fig. 7:**
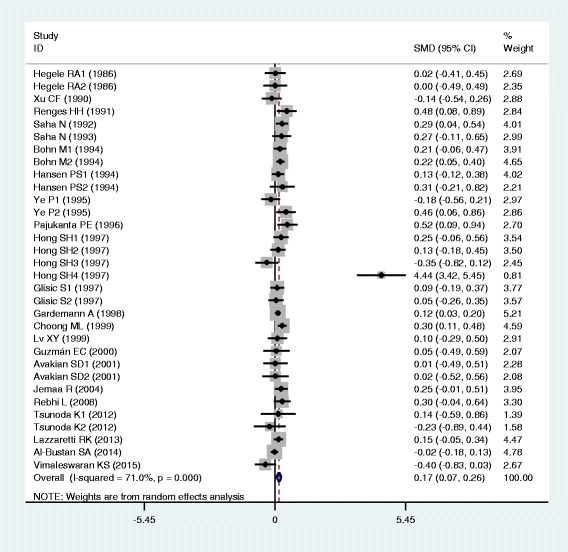
Forest plot of the meta-analysis between *APOB* rs17240441 polymorphism and plasma TC levels (32 comparisons and 7875 subjects were included)

**Fig. 8:**
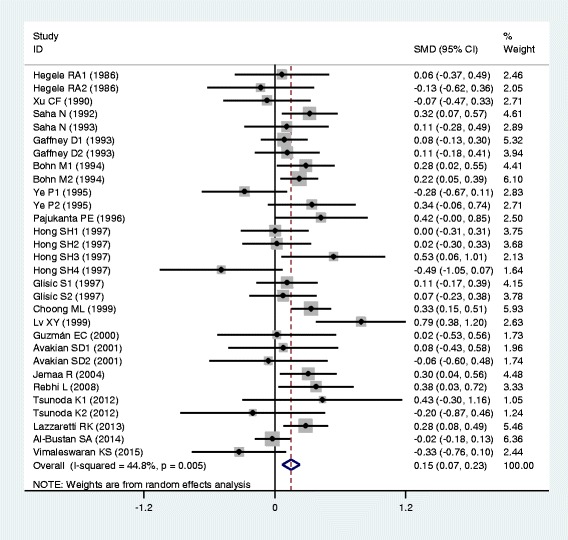
Forest plot of the meta-analysis between *APOB* rs17240441 polymorphism and plasma LDL-C levels (30 comparisons and 5658 subjects were included)

**Fig. 9:**
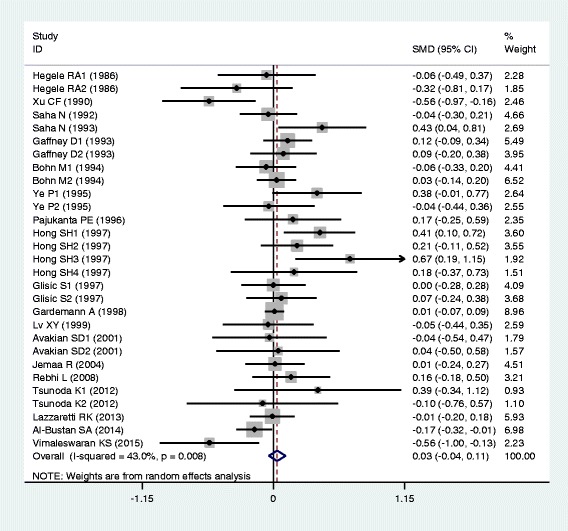
Forest plot of the meta-analysis between *APOB* rs17240441 polymorphism and plasma TG levels (29 comparisons and 7411 subjects were included)

**Fig. 10:**
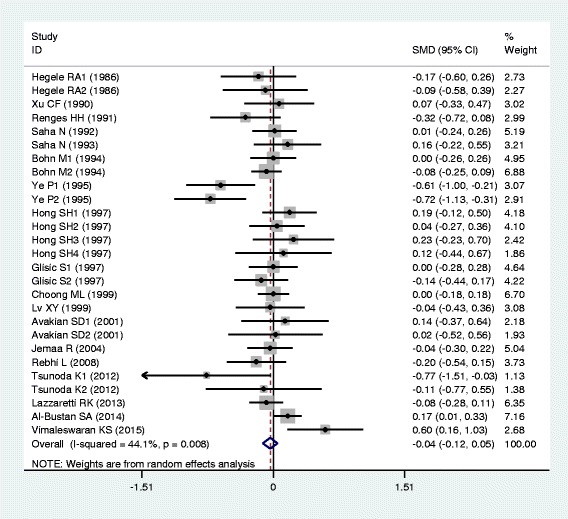
Forest plot of the meta-analysis between *APOB* rs17240441 polymorphism and plasma HDL-C levels (27 comparisons and 5124 subjects were included)

The outcomes of the analyses on all comparisons for the rs17240441 polymorphism showed that the D allele carriers had higher levels of APOB (SMD = 0.13, 95% CI = 0.06–0.20, *P* < 0.01), TC (SMD = 0.17, 95% CI = 0.07–0.26, *P* < 0.01) and LDL-C (SMD = 0.15, 95% CI = 0.07–0.23, *P* < 0.01) than the non-carriers (Table 2, Figs. 6, 7 and 8). There were no significant differences in TG and HDL-C levels between the genotypes (Table 2, Figs. 9 and 10). When the analyses were limited to the studies in HWE, the associations between the rs17240441 polymorphism and higher levels of APOB (SMD = 0.11, 95% CI = 0.04–0.19, *P* < 0.01), TC (SMD = 0.18, 95% CI = 0.04–0.33, *P* = 0.01) and LDL-C (SMD = 0.15, 95% CI = 0.04–0.25, *P* < 0.01) were also significant (Table 2). In the cumulative analyses according to the publication years between the rs17240441 polymorphism and lipid levels, the associations became significant from years 1998, 1992 and 1994 for APOB, TC and LDL-C, respectively (Additional file 1: Figures S25, S27 and S28).

In the subgroup analyses stratified by the characteristics of subjects and the types of studies, the significant association between the rs17240441 polymorphism and higher levels of APOB (SMD = 0.11, 95% CI = 0.03–0.18, *P* < 0.01) was detected only in the CHD patients, but not in the healthy/control subjects. The rs17240441 polymorphism was found to be significantly associated with TG levels (SMD = 0.21, 95% CI = 0.06–0.36, *P* < 0.01) in Asians, but not in Caucasians. The significant association between the rs17240441 polymorphism and higher levels of TC was detected in males (SMD = 0.14, 95% CI = 0.07–0.21, *P* < 0.01), Caucasians (SMD = 0.13, 95% CI = 0.06–0.20, *P* < 0.01), Asians (SMD = 0.33, 95% CI = 0.06–0.60, *P* = 0.02), CHD patients (SMD = 0.12, 95% CI = 0.05–0.19, *P* < 0.01) and the healthy/control subjects (SMD = 0.23, 95% CI = 0.05–0.40, *P* = 0.01). The significant association between the rs17240441 polymorphism and higher levels of LDL-C was detected in both Caucasians (SMD = 0.14, 95% CI = 0.05–0.23, *P* < 0.01) and Asians (SMD = 0.18, 95% CI = 0.00–0.35, *P* = 0.05). The significant association between the rs17240441 polymorphism and higher levels of APOB (SMD = 0.12, 95% CI = 0.03–0.21, *P* < 0.01) was detected only in Caucasians, but not in Asians. The significant association between the rs17240441 polymorphism and higher levels of LDL-C (SMD = 0.13, 95% CI = 0.02–0.24, *P* = 0.02) was detected only in the healthy/control subjects, but not in the CHD patients. When the types of studies were taken into account, the significant associations between the rs17240441 polymorphism and higher levels of APOB (SMD = 0.13, 95% CI = 0.04–0.21, *P* < 0.01), TC (SMD = 0.22, 95% CI = 0.03–0.41, *P* = 0.02) and LDL-C (SMD = 0.12, 95% CI = 0.02–0.23, *P* = 0.03) were detected in the case-control studies. The significant associations between the rs17240441 polymorphism and higher levels of TC (SMD = 0.14, 95% CI = 0.05–0.23, *P* < 0.01) and LDL-C (SMD = 0.18, 95% CI = 0.06–0.31, *P* < 0.01) were detected in the cohort studies.

### Heterogeneity analysis

In the analyses for the rs693 polymorphism, there was significant heterogeneity in the total comparisons for APOB, TG, TC, LDL-C and HDL-C (Table 1). Eight comparisons (Hansen PS 1993, Ye P1 1995, Zhao RZ 1997, Korhonen T 1999, Li S 2003, Hu P 2007, Hu P 2008, Hu P 2009), 7 comparisons (Myant NB2 1989, Pan JP2 1995, Ye P1 1995, Yao J2 1999, Hu P 2007, Srivastava N1 2013, Srivastava N2 2013), 15 comparisons (Bohn M4 1993, Hansen PS 1993, Zhao RZ 1997, Korhonen T 1999, Guzman EC2 2000, Wei JB1 2001, Gong LG1 2003, Li S 2003, Hu P 2007, Hu P 2008, Hu ZY1 2008, Hu ZY2 2008, Hu P 2009, Ma ZZ1 2012, Kulminski AM4 2016), 10 comparisons (Ye P1 1995, Zhao RZ 1997, Guzman EC2 2000, Li S 2003, Gong LG1 2003, Duman BS2 2005, Hu P 2007, Hu P 2008, Hu P 2009, Gallegos-Arreola MP1 2012) and 10 comparisons (Renges HH 1991, Ye P1 1995, Gong LG2 2003, Tan YF2 2003, Gong WD2 2005, Hu ZY1 2008, Hu ZY2 2008, Hu P 2007, Hu P 2009, Kulminski AM4 2016) were respectively identified as the main contributors to the heterogeneity for APOB, TG, TC, LDL-C and HDL-C by using Galbraith plots (Additional file 1: Figures S1-S5). The heterogeneity was effectively removed or decreased after exclusion of these outlier studies, but the SMD values and their 95% CIs did not change substantially (APOB: SMD = 0.16, 95% CI = 0.11–0.20, *P*_SMD_ < 0.01, *P*_Heterogeneity_ = 0.52; TG: SMD = 0.07, 95% CI = 0.03–0.10, *P*_SMD_ < 0.01, *P*_Heterogeneity_ = 0.80; TC: SMD = 0.13, 95% CI = 0.10–0.16, *P*_SMD_ < 0.01, *P*_Heterogeneity_ = 0.31; LDL-C: SMD = 0.14, 95% CI = 0.11–0.18, *P*_SMD_ < 0.01, *P*_Heterogeneity_ = 0.69; HDL-C: SMD = −0.05, 95% CI = −0.08–0.03, *P*_SMD_ < 0.01, *P*_Heterogeneity_ = 0.43).

In the analyses for the rs17240441 polymorphism, there was significant heterogeneity in the total comparisons for TG, TC, LDL-C and HDL-C (Table 2). Four comparisons (Xu CF 1990, Hong SH1 1997, Hong SH3 1997, Vimaleswaran KS 2015), 2 comparisons (Hong SH4 1997, Vimaleswaran KS 2015), 3 comparisons (Hong SH4 1997, Lu XY 1999, Al-Bustan SA 2014) and 4 comparisons (Ye P1 1995, Ye P2 1995, Al-Bustan SA 2014, Vimaleswaran KS 2015) were respectively identified as the main contributors to the heterogeneity for TG, TC, LDL-C and HDL-C by using Galbraith plots (Additional file 1: Figures S6-S9). The heterogeneity was effectively removed or decreased after exclusion of these outlier studies, but the SMD values and their 95% CIs did not change substantially (TG: SMD = 0.02, 95% CI = −0.03−0.07, *P*_SMD_ = 0.51, *P*_Heterogeneity_ = 0.62; TC: SMD = 0.15, 95% CI = 0.09–0.20, *P*_SMD_ < 0.01, *P*_Heterogeneity_ = 0.31; LDL-C: SMD = 0.16, 95% CI = 0.09–0.23, *P*_SMD_ < 0.01, *P*_Heterogeneity_ = 0.16; HDL-C: SMD = −0.03, 95% CI = −0.10−0.03, *P*_SMD_ = 0.35, *P*_Heterogeneity_ = 0.91).

### Publication bias test

For the rs693 polymorphism, no publication bias was detected in the analyses for APOB, TG, LDL-C and HDL-C (Additional file 1: Figures S10, S11, S13 and S14). However, there might be a publication bias in the pooling analysis for TC (*Z* = 1.97, *P* = 0.05) (Additional file 1: Figure S12). To clarify this problem, a trim-and-fill method was employed to adjust the result, and no trimming was performed and the result was unchanged. It indicated that there was no publication bias for TC analysis. The significant *P* value of the Begg’s test was originated from other factors, e.g., heterogeneity. In the present study, Begg’s test did not find any publication bias in the association analyses between the rs17240441 polymorphism and lipids (Additional file 1: Figures S15-S19).

## Discussion

A substantial body of literature has investigated the associations of the rs693 and rs17240441 polymorphisms with plasma APOB and/or lipid levels [4, 6, 8–81]. Associations of the two polymorphisms with increased levels of APOB, TG, TC and LDL-C, and/or decreased levels of HDL-C have been reported in some, but not all studies. The lack of consistency across these studies reflects some existed limitations such as small sample size, ethnic difference and research methodology. In the present study, the associations of the *APOB* rs693 and rs17240441 polymorphisms with plasma APOB and lipid levels were investigated by meta-analysis to clarify these discrepancies.

In most of the included studies, a dominant model was adopted for analysis, i.e., CC vs. CT + TT for the rs693 polymorphism; II vs. ID + DD for the rs17240441 polymorphism. Therefore, a dominant model was also employed for both of the polymorphisms in this meta-analysis to ensure adequate statistical power. Our results suggested that the rs693 polymorphism was significantly associated with higher levels of APOB, TG, TC and LDL-C, and lower levels of HDL-C in the total population. In subgroup analyses, we found that the lipid level differences between genotypes were mainly from Asian populations, in which the SMD values for APOB, TG, TC, LDL-C and HDL-C were bigger when compared to those from Caucasian populations (Table 1). A meta-analysis [82] demonstrated no significant association between the rs693 polymorphism and CHD in the populations involved various ethnicities. However, another meta-analysis [83] revealed that the rs693 polymorphism was associated with higher risk of CHD in the non-Caucasian populations, but not in Caucasian populations. A recent meta-analysis by Chen et al. [5] reported that the rs693 polymorphism was associated with higher risk of CHD in Asian Chinese. In combination with our findings, it is possible that the association between the rs693 polymorphism and CHD in Asians is mediated by increased atherogenic lipid levels (TG, TC and LDL-C) and/or decreased HDL-C levels caused by the T allele of the rs693 polymorphism, since hypertriglyceridemia, hypercholesterolemia and hypo-HDL cholesterolemia are all recognized risk factors for CHD. In the subgroup analyses stratified by the ethnicity of subjects, we found that the rs693 polymorphism was significantly associated with higher levels of TG, TC and LDL-C, but not HDL-C in Asians, which is consistent with the recent results obtained from another meta-analysis specifically on Asian Chinese [84]. The present meta-analysis also demonstrated a significant association between the rs17240441 polymorphism and higher levels of APOB, TC and LDL-C, which explains why the rs17240441 polymorphism was associated with a higher risk of CHD in recent meta-analyses [82, 83].

Subgroup analyses by gender, ethnicity and health condition were performed since they might be important variables in determining associative risk with lipid levels. For example, the present meta-analysis indicated that gender might modulate the associations of the rs693 polymorphism with TC and LDL-C levels since the significant associations only existed in males, but not in females (Table 1). Ethnicity might modulate the associations of the rs693 polymorphism with TG levels because the significant associations only existed in Asians, but not in Caucasians (Table 1). Health status might also modulate the associations between the rs693 polymorphism and APOB and LDL-C levels in that the significant associations only existed in the healthy/control subjects, but not in CHD patients (Table 1). The associations of the rs693 polymorphism with plasma levels of APOB and lipids were very robust, which did not vary greatly when the analyses were performed only with the available studies in HWE. Regarding the rs17240441 polymorphism, the results indicated that gender might modulate the association between the rs17240441 polymorphism and TC levels since the significant association only existed in males, but not in females (Table 2). Ethnicity might modulate the associations of the rs17240441 polymorphism with APOB and TG levels, i.e., the significant effect of the rs17240441 polymorphism on TG only existed in Asians, but not in Caucasians; the significant effect of the rs17240441 polymorphism on APOB only existed in Caucasians, but not in Asians (Table 2). Health status might also modulate the associations of the rs17240441 polymorphism with APOB and LDL-C levels. The significant association between the rs17240441 polymorphism and APOB only existed in CHD patients, but not in the healthy/control subjects; the significant association between the rs17240441 polymorphism and LDL-C only existed in the healthy/control subjects, but not in CHD patients (Table 2). After exclusion of the studies not in HWE, the significant associations of the rs17240441 polymorphism with APOB, TC and LDL-C were not substantially changed, which indicated that the results were convincing. Further studies are required to examine the associations of the rs693 and rs17240441 polymorphisms with APOB and lipid levels regarding the different effects by gender, ethnicity and health status.

The possible mechanisms by which the rs693 and rs17240441 polymorphisms influence the plasma APOB and lipid levels have not been clarified yet. One possible explanation is that the rare alleles of the two polymorphisms (i.e., rs693 T and rs17240441 D) enhance the transcriptional activity of *APOB* gene, affect the *APOB* mRNA structure, and increase the plasma levels of APOB protein. Some rare alleles in apolipoprotein genes have been reported to enhance the transcriptional activity of the genes and accordingly increased the plasma levels of apolipoproteins [85, 86]. Our data in Table 1 and Table 2 have shown that the T allele carriers of the rs693 polymorphism and the D allele carriers of the rs17240441 polymorphism had higher levels of plasma APOB than the non-carriers. The expression of the *APOB* gene is regulated by liver X receptor alpha (LXRα) [87] and MAPK(erk) [88], but not SREBP1 [89]. *APOB* gene is predominantly expressed in small intestine (APOB48) and liver (APOB100). APOB48 is the primary apolipoprotein of chylomicrons which are assembled in small intestine, and APOB100 is the primary apolipoprotein of VLDL particles which are assembled in liver. Chylomicrons and VLDL are two types of large lipoprotein particles which are responsible for carrying lipids (triglycerides and cholesterol) from small intestine and liver to the tissues and organs all over the body. In the circulation, higher levels of APOB lead to higher levels of chylomicron and VLDL particles, and accordingly higher levels of TG and TC. LDL particles are formed in the bloodstream as VLDL particles lose TG through the action of lipoprotein lipase (LPL). Hence, the increase of VLDL levels can cause the elevation of LDL levels, and accordingly the LDL-C levels. There is a profound relationship between plasma levels of TG and HDL-C. In many persons, a higher plasma level of TG correlates with a lower level of HDL-C [90].

Significant heterogeneity was detected in the analyses for the rs693 polymorphism (APOB, TG, TC, LDL-C and HDL-C) and the rs17240441 polymorphism (TG, TC, LDL-C and HDL-C). Subgroup analyses stratified by the characteristics of the subjects were performed to explore the potential sources of the observed heterogeneity, and the results showed that the main sources of heterogeneity were from gender, health condition and ethnic origin of the subjects. The classification of ethnicity of the included studies was divided into Caucasians, Asians and the populations of other ethnic origins. The populations of other ethnic origins were very diverse, including Jewish people, Brazilians, Turkish people, Mexicans, Kuwaitis and Africans. Galbraith plots were employed to figure out the specific comparisons which produced heterogeneity. Outlier comparisons were identified by using the Galbraith plots, and the heterogeneity was effectively removed or decreased after exclusion of the outlier comparisons. No significant changes in the SMD values and 95% CIs were found after excluding the outlier studies.

The associations of the *APOB* rs693 and rs17240441 polymorphisms with plasma APOB and lipids were not likely to be type I errors (false-positive results). Firstly, the results from this meta-analysis were based on the random effects model. Comparing with fixed effects model, the random effects model is a more conservative method and less likely to produce false-positive results. Secondly, 50,018 subjects and 8425 subjects were respectively included in the analyses for the rs693 and rs17240441 polymorphisms. Among the subjects, 55.0% (rs693) and 51.1% (rs17240441) of them were respectively the carriers of the variant allele. Since the incidence of the variant allele carriers was very high, type I error could have been prevented for both of the polymorphisms.

The present meta-analysis has several limitations. Firstly, dyslipidaemia is involved in a large number of genes as well as some environmental factors. However, the interactions of the rs693 and rs17240441 polymorphisms with other polymorphic loci or environmental factors on plasma APOB and lipid levels have not been investigated in this analysis due to the lack of the original data from the included studies. In other words, more precise results could have been gained if more detailed individual data were available, or the stratification analyses based on the environmental factors such as diet, exercise, smoking status, etc., were performed. Secondly, a relatively small number of subjects were included in the association analyses for the rs17240441 polymorphism due to the limited studies that met the inclusion criteria, which may reduce the statistic power and even cause type II error (false-negative results). Another reason for the small number of subjects included in the analyses for the rs17240441 polymorphism might be the change of the rs number from rs11279109 to rs17240441, resulting in missing some relevant articles. Thirdly, this meta-analysis only included the studies published in English and Chinese as it was very difficult to get the full papers published in various languages.

## Conclusions

In conclusion, the significant associations between the rs693 polymorphism and higher levels of APOB, TG, TC and LDL-C, and lower levels of HDL-C were detected in the present meta-analysis. In addition, the significant associations between the rs17240441 polymorphism and higher levels of APOB, TC and LDL-C were also found.

## Methods

### Identification and eligibility of relevant studies

All articles published before December 2016 on the associations of the *APOB* rs693 and/or rs17240441 polymorphisms with plasma APOB and lipid levels were identified. The languages of the articles were limited to English and Chinese. A comprehensive search of the literature was carried out by using the databases including Medline, Google Scholar, Web of Science, Embase, Cochrane Library, Wanfang, VIP and CNKI databases. The keywords used for this search were “apolipoprotein B-100 or apolipoprotein B100 or apolipoprotein B or APOB-100 or APOB100 or APOB or APO B-100 or APO B100 or APO B” concatenated with “polymorphism or variant or mutation or SNP”. The variables of this meta-analysis were limited to APOB, TG, TC, LDL-C and HDL-C. The studies that fulfilled the following criteria were included: (1) studies in which mean lipids and standard deviations (SD) or standard errors (SE) by the rs693 or rs17240441 genotypes were available; (2) data reported on APOB and/or at least one of the four plasma lipid variables; (3) data reported on fasting lipid variables; (4) pre-intervention baseline data were used for interventional studies. All references cited by the included articles were reviewed to check the published work which was not indexed by Medline, Google Scholar, Web of Science, Embase, Cochrane Library, Wanfang, VIP or CNKI database. Reports with incomplete data, studies based on pedigree data, case reports, review articles, abstracts and animal studies were excluded from the meta-analysis.

### Data extraction

Data were extracted by using a structured data collection form. The irrelevant studies or the studies that did not meet the inclusion criteria were excluded after being reviewed independently by two reviewers. The data were double-checked and compared after extraction. The uncertainty in the data was discussed and solved by the whole group. For the overlapping articles, only the publications that presented the most detailed information were included. In the present meta-analysis, the data extracted from each of the included studies were as follows: first author, year of publication, age, ethnicity, gender, health condition, genotyping and lipid assay methods, sample size, mean APOB or lipid variables and SD or SE by genotypes.

### Statistical analysis

The STATA software package (Version 10, Stata Corporation, College Station, TX) was used in the meta-analysis. All data were presented as mean ± SD in this meta-analysis. For the articles in which mean ± SE was given, the value of the SD was calculated. The units g/L and mmol/L were respectively used for APOB and lipids in the meta-analysis, and unit conversion was conducted for the articles in which other units were used. HWE of the populations was tested by χ^2^ test, and the significance level was defined as α < 0.05. Since most of the included studies reported the results in a dominant way [i.e., CC vs. (CT + TT) for rs693; II vs. (ID + DD) for rs17240441], a dominant model was employed in the present meta-analysis to ensure adequate statistical power. When data were presented for more than one subpopulation (e.g., male or female subjects, the subjects from different ethnicity, or the subjects with different health status) in one article, each subpopulation was treated as a separate comparison in this meta-analysis. Subgroup analyses were conducted according to gender, ethnicity, health condition and type of study. Ethnicity was defined as Caucasian, Asian, and the populations of other ethnic origins. Health condition was defined as healthy/control subjects, CHD patients, diabetic patients, etc. Type of study was defined as case-control study and cohort study. The subgroup analyses were performed with at least 4 comparisons to ensure adequate statistical power. Cumulative analyses were conducted to guarantee the strength of results.

The random effects model was used in the meta-analysis in that (1) both between-study and within-study heterogeneity is considered in random effects model; (2) the random effects model provides a more conservative evaluation of the significance of the associations than the fixed effects model [91]. The SMD and 95% CI were used to assess the differences in APOB and lipid levels between the genotypes. Heterogeneity among studies was tested by Cochran’s χ^2^-based Q-statistic at a significance level of *P* < 0.05. Galbraith plots were used to detect the potential sources of heterogeneity, and the SMD values were recalculated after excluding the outlier comparisons. Publication bias was assessed by Begg’s rank correlation tests [92] and funnel plots, and a significance level of 0.05 was used to assess the presence of potential publication bias.

## Additional file

Additional file 1: Table S1. Characteristics of the studies included in the meta-analysis for the rs693 and rs17240441 polymorphisms; **Table S2.** Plasma APOB and lipid levels by the rs693 genotypes of the individual studies included in the meta-analysis; **Table S3.** Plasma APOB and lipid levels by the rs17240441 genotypes of the individual studies included in the meta-analysis; **Figures S1-S5.** Galbraith plots for the association analyses between the *APOB* rs693 polymorphism and APOB, TG, TC, LDL-C and HDL-C, respectively; **Figures S6-S9.** Galbraith plots for the association analyses between the *APOB* rs17240441 polymorphism and TG, TC, LDL-C and HDL-C, respectively; **Figures S10-S14.** Begg’s funnel plots for the association analyses between the *APOB* rs693 polymorphism and APOB, TG, TC, LDL-C and HDL-C, respectively; **Figures S15-S19.** Begg’s funnel plot for the association analyses between the APOB rs17240441 polymorphism and APOB, TG, TC, LDL-C and HDL-C respectively. **Figures S20-S24.** Cumulative analysis plots according to the publication years for the association analyses between the APOB rs693 polymorphism and APOB, TG, TC, LDL-C and HDL-C respectively. **Figures S25-S29.** Cumulative analysis plots according to the publication years for the association analyses between the APOB rs17240441 polymorphism and APOB, TG, TC, LDL-C and HDL-C respectively. (DOC 573 kb)

## Abbreviations

95% CI: 95% confidence interval
ApoAI: Apolipoprotein AI
ApoB: Apolipoprotein B
BMI: Body mass index
CHD: Coronary heart disease
HDL-C: High-density lipoprotein cholesterol
LDL-C: Low-density lipoprotein cholesterol
SMD: Standardized mean difference
SNP: Single nucleotide polymorphism
TC: Total cholesterol
TG: Triglycerides
