# Supplementary material for: Associations of the APOB rs693 and rs17240441 polymorphisms with plasma APOB and lipid levels: a meta-analysis
Source: Lipids Health Dis. 2017 Sep 6;16:166. doi: 10.1186/s12944-017-0558-7 (PMC5586014; doi:10.1186/s12944-017-0558-7)
Supplement: Additional file 1: Table S1. — Characteristics of the studies included in the meta-analysis for the rs693 and rs17240441 polymorphisms; Table S2. Plasma APOB and lipid levels by the rs693 genotypes of the individual studies included in the meta-analysis; Table S3. Plasma APOB and lipid levels by the rs17240441 genotypes of the individual studies included in the meta-analysis; Figures S1-S5. Galbraith plots for the association analyses between the APOB rs693 polymorphism and APOB, TG, TC, LDL-C and HDL-C, respectively; Figures S6-S9. Galbraith plots for the association analyses between the APOB rs17240441 polymorphism and TG, TC, LDL-C and HDL-C, respectively; Figures S10-S14. Begg’s funnel plots for the association analyses between the APOB rs693 polymorphism and APOB, TG, TC, LDL-C and HDL-C, respectively; Figures S15-S19. Begg’s funnel plot for the association analyses between the APOB rs17240441 polymorphism and APOB, TG, TC, LDL-C and HDL-C respectively. Figures S20-S24. Cumulative analysis plots according to the publication years for the association analyses between the APOB rs693 polymorphism and APOB, TG, TC, LDL-C and HDL-C respectively. Figures S25-S29. Cumulative analysis plots according to the publication years for the association analyses between the APOB rs17240441 polymorphism and APOB, TG, TC, LDL-C and HDL-C respectively. (DOC 573 kb) [file 12944_2017_558_MOESM1_ESM.doc]

**Supplemental Tables:**

**Table S1:** Characteristics of the studies included in the meta-analysis for the rs693 and rs17240441 polymorphisms

**Table S2:** Plasma APOB and lipid levels by the rs693 genotypes of individual studies included in the meta-analysis

**Table S3:** Plasma APOB and lipid levels by the rs17240441 genotypes of individual studies included in the meta-analysis

**Supplemental Figures:**

**Figure S1:** Galbraith plot for the association analysis between the *APOB* rs693 polymorphism and APOB

**Figure S2:** Galbraith plot for the association analysis between the *APOB* rs693 polymorphism and TG

**Figure S3:** Galbraith plot for the association analysis between the *APOB* rs693 polymorphism and TC

**Figure S4:** Galbraith plot for the association analysis between the *APOB* rs693 polymorphism and LDL-C

**Figure S5:** Galbraith plot for the association analysis between the *APOB* rs693 polymorphism and HDL-C

**Figure S6:** Galbraith plot for the association analysis between the *APOB* rs17240441 polymorphism and TG

**Figure S7:** Galbraith plot for the association analysis between the *APOB* rs17240441 polymorphism and TC

**Figure S8:** Galbraith plot for the association analysis between the *APOB* rs17240441 polymorphism and LDL-C

**Figure S9:** Galbraith plot for the association analysis between the *APOB* rs17240441 polymorphism and HDL-C

**Figure S10:** Begg’s funnel plot for the association analysis between the *APOB* rs693 polymorphism and APOB

**Figure S11:** Begg’s funnel plot for the association analysis between the *APOB* rs693 polymorphism and TG

**Figure S12:** Begg’s funnel plot for the association analysis between the *APOB* rs693 polymorphism and TC

**Figure S13:** Begg’s funnel plot for the association analysis between the *APOB* rs693 polymorphism and LDL-C

**Figure S14:** Begg’s funnel plot for the association analysis between the *APOB* rs693 polymorphism and HDL-C

**Figure S15:** Begg’s funnel plot for the association analysis between the *APOB* rs17240441 polymorphism and APOB

**Figure S16:** Begg’s funnel plot for the association analysis between the *APOB* rs17240441 polymorphism and TG

**Figure S17:** Begg’s funnel plot for the association analysis between the *APOB* rs17240441 polymorphism and TC

**Figure S18:** Begg’s funnel plot for the association analysis between the *APOB* rs17240441 polymorphism and LDL-C

**Figure S19:** Begg’s funnel plot for the association analysis between the *APOB* rs17240441 polymorphism and HDL-C

**Figure S20:** Cumulative analysis plot according to publication years for the association analysis between the *APOB* rs693 polymorphism and APOB.

**Figure S21:** Cumulative analysis plot according to the publication years for the association analysis between the *APOB* rs693 polymorphism and TG.

**Figure S22:** Cumulative analysis plot according to the publication years for the association analysis between the *APOB* rs693 polymorphism and TC.

**Figure S23:** Cumulative analysis plot according to the publication years for the association analysis between the *APOB* rs693 polymorphism and LDL-C.

**Figure S24:** Cumulative analysis plot according to the publication years for the association analysis between the *APOB* rs693 polymorphism and HDL-C.

**Figure S25:** Cumulative analysis plot according to the publication years for the association analysis between the *APOB* rs17240441 polymorphism and APOB.

**Figure S26:** Cumulative analysis plot according to the publication years for the association analysis between the *APOB* rs17240441 polymorphism and TG.

**Figure S27:** Cumulative analysis plot according to the publication years for the association analysis between the *APOB* rs17240441 polymorphism and TC.

**Figure S28:** Cumulative analysis plot according to the publication years for the association analysis between the *APOB* rs17240441 polymorphism and LDL-C.

**Figure S29:** Cumulative analysis plot according to the publication years for the association analysis between the *APOB* rs17240441 polymorphism and HDL-C.

**Table S1:** Characteristics of the studies included in the meta-analyses for the rs693 and rs17240441 polymorphisms.

| First author, reference | Polymorphisms | year | Ethnicity | Gender | Study population | Outcomes |
| --- | --- | --- | --- | --- | --- | --- |
| Hegele RA1 [47] | rs17240441 | 1986 | Caucasian | M/F | CHD patients | APOB, TG, TC, LDL-C, HDL-C |
| Hegele RA2 [47] | rs17240441 | 1986 | Caucasian | M/F | Control subjects | APOB, TG, TC, LDL-C, HDL-C |
| Talmud PJ [19] | rs693 | 1987 | Caucasian | M/F | Healthy subjects | TG, TC, LDL-C |
| Aalto-Setala K1 [48] | rs693 | 1988 | Caucasian | M | Healthy subjects | APOB, TG, TC, LDL-C, HDL-C |
| Aalto-Setala K2 [48] | rs693 | 1988 | Caucasian | F | Healthy subjects | APOB, TG, TC, LDL-C, HDL-C |
| Myant NB1 [33] | rs693 | 1989 | Caucasian | M | CHD patients | APOB, TG, TC, LDL-C, HDL-C |
| Myant NB2 [33] | rs693 | 1989 | Caucasian | M | Control subjects | APOB, TG, TC, LDL-C, HDL-C |
| Darnfors C1 [49] | rs693 | 1989 | Caucasian | M | Healthy subjects | APOB, TG, TC, LDL-C, HDL-C |
| Darnfors C2 [49] | rs693 | 1989 | Caucasian | F | Healthy subjects | APOB, TG, TC, LDL-C, HDL-C |
| Paulweber B1 [23] | rs693 | 1990 | Caucasian | M | CHD patients | TG, TC, LDL-C |
| Paulweber B2 [23] | rs693 | 1990 | Caucasian | M | Control subjects | TG, TC, LDL-C |
| Genest JJ Jr [50] | rs693 | 1990 | Caucasian | M | CHD patients | APOB, TG, TC, LDL-C, HDL-C |
| Xu CF [43] | rs17240441 | 1990 | Caucasian | M/F | Healthy subjects | APOB, TG, TC, LDL-C, HDL-C |
| Aalto-Setala K [32] | rs693 | 1991 | Caucasian | M | Dyslipidemic subjects | TG, TC, LDL-C, HDL-C |
| Renges HH [34] | rs693, rs17240441 | 1991 | Asian | M | Healthy subjects | TC, HDL-C |
| Vilella E [24] | rs693 | 1992 | Caucasian | M | Healthy subjects | APOB, TG, TC, LDL-C, HDL-C |
| Saha N [44] | rs17240441 | 1992 | Asian | M/F | Healthy subjects | APOB, TG, TC, LDL-C, HDL-C |
| Saha N1 [35] | rs693 | 1992 | Asian | M/F | CHD patients | APOB, TG, TC, LDL-C, HDL-C |
| Saha N2 [35] | rs693 | 1992 | Asian | M/F | Healthy subjects | APOB, TG, TC, LDL-C, HDL-C |
| Saha N1 [51] | rs693 | 1992 | Asian | M | Healthy subjects | APOB, TG, TC, LDL-C, HDL-C |
| Saha N2 [51] | rs693 | 1992 | Asian | F | Healthy subjects | APOB, TG, TC, LDL-C, HDL-C |
| Saha N [52] | rs693, rs17240441 | 1993 | Asian | M/F | Healthy subjects | APOB, TG, TC, LDL-C, HDL-C |
| Gaffney D1 [59] | rs17240441 | 1993 | Caucasian | M | Mild hypercholesterolaemia | TG, LDL-C |
| Gaffney D2 [59] | rs17240441 | 1993 | Caucasian | M/F | Healthy subjects | TG, LDL-C |
| Evans AE [58] | rs693 | 1993 | Asian | M | Healthy subjects | APOB, TG, LDL-C, HDL-C |
| Friedlander Y1 [10] | rs693 | 1993 | Jewish | M | Healthy subjects | APOB, TG, TC, LDL-C, HDL-C |
| Friedlander Y2 [10] | rs693 | 1993 | Jewish | F | Healthy subjects | APOB, TG, TC, LDL-C, HDL-C |
| Hansen PS [17] | rs693 | 1993 | Caucasian | M | Healthy subjects | APOB, TC |
| Ukkola O [20] | rs693 | 1993 | Caucasian | M/F | Diabetic patients | TG, TC, LDL-C, HDL-C |
| Bohn M1 [11] | rs693 | 1993 | Caucasian | M | CHD patients | APOB, TG, TC, LDL-C, HDL-C |
| Bohn M2 [11] | rs693 | 1993 | Caucasian | M | Control subjects | APOB, TG, TC, LDL-C, HDL-C |
| Bohn M3 [11] | rs693 | 1993 | Caucasian | F | CHD patients | APOB, TG, TC, LDL-C, HDL-C |
| Bohn M4 [11] | rs693 | 1993 | Caucasian | F | Control subjects | APOB, TG, TC, LDL-C, HDL-C |
| Hansen PS1 [61] | rs693, rs17240441 | 1994 | Caucasian | M | CHD patients | TC |
| Hansen PS2 [61] | rs693, rs17240441 | 1994 | Caucasian | F | CHD patients | TC |
| Bohn M1 [45] | rs17240441 | 1994 | Caucasian | M/F | CHD patients | APOB, TG, TC, LDL-C, HDL-C |
| Bohn M2 [45] | rs17240441 | 1994 | Caucasian | M/F | Control subjects | APOB, TG, TC, LDL-C, HDL-C |
| Gajra B [60] | rs693 | 1994 | Asian | M/F | Healthy subjects | TG, TC, LDL-C |
| Ye P1 [36] | rs693, rs17240441 | 1995 | Asian | M/F | CHD patients | APOB, TG, TC, LDL-C, HDL-C |
| Ye P2 [36] | rs693, rs17240441 | 1995 | Asian | M/F | Control subjects | APOB, TG, TC, LDL-C, HDL-C |
| Pan JP1 [53] | rs693 | 1995 | Asian | M/F | CHD patients | APOB, TG, TC, LDL-C, HDL-C |
| Pan JP2 [53] | rs693 | 1995 | Asian | M/F | Control subjects | APOB, TG, TC, LDL-C, HDL-C |
| Pajukanta PE [38] | rs693, rs17240441 | 1996 | Caucasian | M/F | Healthy subjects | APOB, TG, TC, LDL-C |
| Guo Y [73] | rs693 | 1996 | Asian | M/F | Ischemic cerebrovascular patients | TG, TC, LDL-C, HDL-C |
| Feng JS1 [72] | rs693 | 1997 | Asian | M/F | Healthy subjects | TG, TC, |
| Feng JS2 [72] | rs693 | 1997 | Asian | M/F | Hypercholesterolemia | TG, TC, |
| Feng JS3 [72] | rs693 | 1997 | Asian | M/F | Hypertriglyceridemia | TG, TC, |
| Zhao RZ [70] | rs693 | 1997 | Asian | M/F | Cerebral infarction patients | APOB, TG, TC, LDL-C, HDL-C |
| Corbo RM1 [27] | rs693 | 1997 | Caucasian | F | CHD patients | TC |
| Corbo RM2 [27] | rs693 | 1997 | Caucasian | F | Control subjects | TC |
| Hong SH1 [41] | rs17240441 | 1997 | Asian | M | CHD patients | TG, TC, LDL-C, HDL-C |
| Hong SH2 [41] | rs17240441 | 1997 | Asian | M | Control subjects | TG, TC, LDL-C, HDL-C |
| Hong SH3 [41] | rs17240441 | 1997 | Asian | F | CHD patients | TG, TC, LDL-C, HDL-C |
| Hong SH4 [41] | rs17240441 | 1997 | Asian | F | Control subjects | TG, TC, LDL-C, HDL-C |
| Glisic S1 [62] | rs17240441 | 1997 | Caucasian | M | Healthy subjects | TG, TC, LDL-C, HDL-C |
| Glisic S2 [62] | rs17240441 | 1997 | Caucasian | F | Healthy subjects | TG, TC, LDL-C, HDL-C |
| Gardemann A [39] | rs17240441 | 1998 | Caucasian | M | CHD patients | APOB, TG, TC |
| Hubacek JA1 [54] | rs693 | 1998 | Caucasian | M/F | Hypercholesterolemia | APOB, TG, TC, LDL-C, HDL-C |
| Hubacek JA2 [54] | rs693 | 1998 | Caucasian | M/F | Control subjects | APOB, TG, TC, LDL-C, HDL-C |
| Stepanov VA1 [63] | rs693 | 1998 | Caucasian | M | CHD patients | TG, TC |
| Stepanov VA2 [63] | rs693 | 1998 | Caucasian | M | Control subjects | TG, TC, HDL-C |
| Lu XY [81] | rs17240441 | 1999 | Asian | M/F | Healthy subjects | APOB, TG, TC, LDL-C, HDL-C |
| Yao J1 [71] | rs693 | 1999 | Asian | M/F | Cerebral infarction patients | APOB, TG, TC, LDL-C, HDL-C |
| Yao J2 [71] | rs693 | 1999 | Asian | M/F | Control subjects | APOB, TG, TC, LDL-C, HDL-C |
| Choong ML [40] | rs17240441 | 1999 | Asian | M/F | Healthy subjects | APOB, TC, LDL-C, HDL-C |
| Corbo RM1 [37] | rs693 | 1999 | Caucasian | M | CHD patients | HDL-C |
| Corbo RM2 [37] | rs693 | 1999 | Caucasian | M | Control subjects | HDL-C |
| Korhonen T [18] | rs693 | 1999 | Caucasian | M/F | Hypercholesterolemia | APOB, TG, TC |
| Choong ML [64] | rs693 | 1999 | Asian | M/F | Healthy subjects | APOB, TC, LDL-C, HDL-C |
| Wang LY1 [8] | rs693 | 1999 | Asian | M/F | Cerebral infarction | APOB, TG, TC, LDL-C, HDL-C |
| Wang LY2 [8] | rs693 | 1999 | Asian | M/F | Control subjects | APOB, TG, TC, LDL-C, HDL-C |
| Guzman EC1 [25] | rs693, rs17240441 | 2000 | Caucasian | M | High-risk for CHD | TC, LDL-C |
| Guzman EC2 [25] | rs693, rs17240441 | 2000 | Caucasian | F | High-risk for CHD | TC, LDL-C |
| Han T1 [12] | rs693 | 2000 | Asian | M/F | Gallstone patients | APOB, TG, TC, LDL-C, HDL-C |
| Han T2 [12] | rs693 | 2000 | Asian | M/F | Control subjects | APOB, TG, TC, LDL-C, HDL-C |
| Lopez-Miranda J [28] | rs693 | 2000 | Caucasian | M/F | Healthy subjects | APOB, TG, TC, LDL-C, HDL-C |
| Wei JB1 [55] | rs693 | 2001 | Asian | M/F | Gallstone patients | APOB, TG, TC, LDL-C, HDL-C |
| Wei JB2 [55] | rs693 | 2001 | Asian | M/F | Control subjects | APOB, TG, TC, LDL-C, HDL-C |
| Zhu WL1 [56] | rs693 | 2001 | Asian | M | Healthy subjects | APOB, TG, TC, LDL-C, HDL-C |
| Zhu WL2 [56] | rs693 | 2001 | Asian | F | Healthy subjects | APOB, TG, TC, LDL-C, HDL-C |
| Avakian SD1 [65] | rs693, rs17240441 | 2001 | Brazilian | M/F | CHD patients | TG, TC, LDL-C, HDL-C |
| Avakian SD2 [65] | rs693, rs17240441 | 2001 | Brazilian | M/F | Control subjects | TG, TC, LDL-C, HDL-C |
| Baroni MG [9] | rs693 | 2003 | Caucasian | M/F | CHD patients | APOB, TG, TC, LDL-C, HDL-C |
| Li S [13] | rs693 | 2003 | Asian | M/F | Healthy subjects | APOB, TG, TC, LDL-C, HDL-C |
| Gong LG1 [80] | rs693 | 2003 | Asian | M/F | Hypertriglyceridemia | APOB, TG, TC, LDL-C, HDL-C |
| Gong LG2 [80] | rs693 | 2003 | Asian | M/F | Control subjects | APOB, TG, TC, LDL-C, HDL-C |
| Tan YF1 [77] | rs693 | 2003 | Asian | M/F | Gallstone patient | APOB, TG, TC, LDL-C, HDL-C |
| Tan YF2 [77] | rs693 | 2003 | Asian | M/F | Control subjects | APOB, TG, TC, LDL-C, HDL-C |
| Jemaa R [46] | rs17240441 | 2004 | Caucasian | M/F | Overweight subjects | APOB, TG, TC, LDL-C, HDL-C |
| Gong WD1 [79] | rs693 | 2005 | Asian | M/F | Gallstone patients | APOB, TG, TC, LDL-C, HDL-C |
| Gong WD2 [79] | rs693 | 2005 | Asian | M/F | Control subjects | APOB, TG, TC, LDL-C, HDL-C |
| Duman BS1 [66] | rs693 | 2005 | Turkish | M/F | CHD patients | TG, TC, LDL-C, HDL-C |
| Duman BS2 [66] | rs693 | 2005 | Turkish | M/F | Control subjects | TG, TC, LDL-C, HDL-C |
| Duman BS1 [67] | rs693 | 2006 | Turkish | M/F | Diabetic patients | APOB, TG, TC |
| Duman BS2 [67] | rs693 | 2006 | Turkish | M/F | Control subjects | APOB, TG, TC |
| Nieminen T [14] | rs693 | 2007 | Caucasian | M/F | Healthy subjects | APOB, TG, TC, LDL-C, HDL-C |
| Hu P [75] | rs693 | 2007 | Asian | M/F | Healthy subjects | APOB, TG, TC, LDL-C, HDL-C |
| Rebhi L [6] | rs17240441 | 2008 | African | M/F | CHD patients | APOB, TG, TC, LDL-C, HDL-C |
| Hu P [78] | rs693 | 2008 | Asian | M/F | Nephropathy patients | APOB, TG, TC, LDL-C, HDL-C |
| Hu ZY1 [30] | rs693 | 2008 | Asian | M/F | Cerebral hemorrhage | APOB, TG, TC, LDL-C, HDL-C |
| Hu ZY2 [30] | rs693 | 2008 | Asian | M/F | Control subjects | APOB, TG, TC, LDL-C, HDL-C |
| Hu P [15] | rs693 | 2009 | Asian | M/F | Healthy children | APOB, TG, TC, LDL-C, HDL-C |
| Qian J [74] | rs693 | 2010 | Asian | M/F | Hyperlipidaemia | TC, LDL-C, HDL-C |
| Park MH [22] | rs693 | 2011 | Asian | M/F | Healthy subjects | TG, LDL-C |
| Ma ZZ1 [76] | rs693 | 2012 | Asian | M/F | hyperlipidaemia | APOB, TG, TC, LDL-C, HDL-C |
| Ma ZZ2 [76] | rs693 | 2012 | Asian | M/F | Control subjects | APOB, TG, TC, LDL-C, HDL-C |
| Tsunoda K1 [21] | rs693, rs17240441 | 2012 | Asian | M | Healthy subjects | TG, TC, LDL-C, HDL-C |
| Tsunoda K2 [21] | rs693, rs17240441 | 2012 | Asian | F | Healthy subjects | TG, TC, LDL-C, HDL-C |
| Gallegos-Arreola MP1 [4] | rs693 | 2012 | Mexican | M/F | CHD patients | TG, TC, LDL-C, HDL-C |
| Gallegos-Arreola MP2 [4] | rs693 | 2012 | Mexican | M/F | Control subjects | TG, TC, LDL-C, HDL-C |
| Lazzaretti RK [26] | rs693, rs17240441 | 2013 | Brazilian | M/F | HIV-1 patients | TG, TC, LDL-C, HDL-C |
| Srivastava N1 [68] | rs693 | 2013 | Asian | M/F | Obese subjects | TG, TC, LDL-C, HDL-C |
| Srivastava N2 [68] | rs693 | 2013 | Asian | M/F | Healthy weight subjects | TG, TC, LDL-C, HDL-C |
| Al-Bustan SA [57] | rs693, rs17240441 | 2014 | Kuwaiti | M/F | Healthy subjects | TG, TC, LDL-C, HDL-C |
| Berkinbayev S [16] | rs693 | 2014 | Asian | M | CHD patients | APOB, TG, TC, LDL-C, HDL-C |
| Liu YL [31] | rs693 | 2014 | Asian | M/F | Healthy subjects | APOB, TG, TC, LDL-C, HDL-C |
| Starcevic JN [69] | rs693 | 2014 | Caucasian | M/F | Diabetic patients | APOB, TC, LDL-C |
| Vimaleswaran KS [42] | rs17240441 | 2015 | Caucasian | M/F | Healthy subjects | TG, TC, LDL-C, HDL-C |
| Kulminski AM1 [29] | rs693 | 2016 | Caucasian | M/F | Atherosclerosis Risk in Communities (ARIC) Study | TC, HDL-C |
| Kulminski AM2 [29] | rs693 | 2016 | Caucasian | M/F | Framingham Heart Study (FHS) | TC, HDL-C |
| Kulminski AM3 [29] | rs693 | 2016 | Caucasian | M/F | Multi-Ethnic Study of Atherosclerosis (MESA) | TC, HDL-C |
| Kulminski AM4 [29] | rs693 | 2016 | Caucasian | M/F | Cardiovascular Health Study (CHS) | TC, HDL-C |
|  |  |  |  |  |  |  |

**Table S2:** Plasma APOB and lipid levels by the rs693 genotypes of individual studies included in the meta-analysis.

| **First author, reference** | **Genotype** | |  | **APOB, g/L** | |  | **TG, mmol/L** | |  | **TC, mmol/L** | |  | **LDL-C, mmol/L** | |  | **HDL-C, mmol/L** | |
| --- | --- | --- | --- | --- | --- | --- | --- | --- | --- | --- | --- | --- | --- | --- | --- | --- | --- |
|  | CC | CT+TT |  | CC | CT+TT |  | CC | CT+TT |  | CC | CT+TT |  | CC | CT+TT |  | CC | CT+TT |
| Talmud PJ [19] | 12 | 50 |  | - | - |  | 1.15±0.91 | 1.16±0.78 |  | 4.81±0.91 | 5.40±0.66 |  | 2.84±0.95 | 3.30±0.76 |  | - | - |
| Aalto-Setala K1 [48] | 26 | 47 |  | 0.88±0.25 | 0.98±0.25 |  | 1.25±0.64 | 1.47±0.80 |  | 5.60±1.27 | 6.11±1.08 |  | 3.74±1.05 | 4.15±1.03 |  | 1.55±0.56 | 1.60±0.38 |
| Aalto-Setala K2 [48] | 37 | 66 |  | 0.75±0.23 | 0.82±0.29 |  | 0.92±0.40 | 1.07±0.48 |  | 5.25±1.02 | 5.85±1.49 |  | 3.32±0.99 | 3.74±1.41 |  | 1.78±0.40 | 1.90±0.47 |
| Myant NB1 [33] | 30 | 94 |  | 1.41±0.39 | 1.43±0.38 |  | 2.03±1.47 | 2.33±1.58 |  | 6.96±1.21 | 7.02±1.44 |  | 4.86±1.04 | 4.79±1.00 |  | 1.10±0.30 | 0.95±0.25 |
| Myant NB2 [33] | 23 | 79 |  | 1.19±0.43 | 1.11±0.36 |  | 1.15±0.33 | 0.98±0.23 |  | 6.01±1.56 | 5.98±1.08 |  | 4.18±1.39 | 4.24±1.00 |  | 1.17±0.28 | 1.23±0.32 |
| Darnfors C1 [49] | 27 | 78 |  | 0.99±0.25 | 1.00±0.32 |  | 1.55±1.26 | 1.41±0.79 |  | 6.08±1.14 | 6.27±1.19 |  | 4.78±0.93 | 4.92±1.24 |  | 1.30±0.35 | 1.37±0.35 |
| Darnfors C2 [49] | 11 | 71 |  | 0.89±0.35 | 0.88±0.28 |  | 1.03±0.67 | 1.03±0.60 |  | 5.69±1.27 | 6.03±1.47 |  | 3.77±1.28 | 4.36±1.41 |  | 1.93±0.37 | 1.69±0.46 |
| Paulweber B1 [23] | 27 | 73 |  | - | - |  | 1.52±0.81 | 1.69±0.77 |  | 5.76±1.00 | 5.68±0.92 |  | 4.05±1.06 | 3.97±0.94 |  | - | - |
| Paulweber B2 [23] | 29 | 89 |  | - | - |  | 1.50±0.97 | 1.38±0.73 |  | 5.26±0.86 | 5.34±1.00 |  | 3.36±0.92 | 3.54±0.89 |  | - | - |
| Genest JJ Jr [50] | 28 | 83 |  | 1.09±0.28 | 1.06±0.27 |  | 2.64 ±1.28 | 2.16±1.12 |  | 5.43±1.34 | 5.42±1.22 |  | 3.39±1.06 | 3.56±1.15 |  | 0.78±0.23 | 0.84±0.23 |
| Aalto-Setala K [32] | 61 | 158 |  | - | - |  | 2.07±1.37 | 1.87±0.83 |  | 6.93±0.94 | 7.08±1.07 |  | 4.77±0.91 | 5.02±1.15 |  | 1.22±0.28 | 1.21±0.27 |
| Renges HH [34] | 55 | 52 |  | - | - |  | - | - |  | 5.68±0.89 | 6.16±1.36 |  | - | - |  | 1.39±0.37 | 1.19±0.34 |
| Vilella E [24] | 69 | 159 |  | 0.93±0.34 | 1.00±0.42 |  | 1.47±0.95 | 5.31±1.00 |  | 5.31±1.00 | 5.51±0.99 |  | 3.78±0.83 | 3.90±1.02 |  | 1.16±0.33 | 1.20±0.35 |
| Saha N1 [35] | 118 | 13 |  | 1.19±0.75 | 1.40±0.73 |  | 2.34±1.74 | 6.02±2.47 |  | 6.02±2.47 | 7.03±2.91 |  | 4.28±2.53 | 5.01±3.13 |  | 0.80±0.67 | 0.94±0.75 |
| Saha N2 [35] | 124 | 25 |  | 0.68±0.21 | 0.72±0.18 |  | 1.47±0.44 | 5.31±0.79 |  | 5.31±0.79 | 5.10±0.81 |  | 3.49±0.67 | 3.43±0.81 |  | 1.16±0.34 | 1.02±0.25 |
| Saha N1 [51] | 101 | 26 |  | 0.70±0.23 | 0.75±0.20 |  | 1.24±0.50 | 1.32±0.51 |  | 4.99±0.80 | 4.93±0.97 |  | 3.27±0.80 | 3.31±1.02 |  | 1.12±0.30 | 1.02±0.25 |
| Saha N2 [51] | 62 | 7 |  | 0.64±0.25 | 0.62±0.30 |  | 0.94±0.39 | 0.78±0.26 |  | 5.07±0.79 | 4.62±5.29 |  | 3.23±0.79 | 2.88±0.87 |  | 1.42±0.39 | 1.38±0.48 |
| Saha N [52] | 102 | 59 |  | 0.95±0.60 | 0.97±0.73 |  | 1.59±1.90 | 2.11±2.42 |  | 5.66±2.38 | 5.65±2.66 |  | 3.79±2.30 | 3.78±2.48 |  | 1.08±0.63 | 0.89±0.54 |
| Evans AE [58] | 127 | 16 |  | 0.98±0.25 | 1.03±0.18 |  | 1.94±1.21 | 1.89±1.03 |  | - | - |  | 2.56±0.57 | 2.61±0.47 |  | 1.09±0.28 | 1.06±0.26 |
| Friedlander Y1 [10] | 126 | 150 |  | 0.69±0.20 | 0.74±0.26 |  | 1.22±0.60 | 1.24±0.60 |  | 4.34±0.79 | 4.56±0.97 |  | 2.73±0.73 | 2.94±0.76 |  | 0.97±0.28 | 0.94 ± 0.27 |
| Friedlander Y2 [10] | 99 | 149 |  | 0.60±0.16 | 0.66±0.16 |  | 0.97±0.40 | 0.98±0.43 |  | 4.30±0.82 | 4.50±0.81 |  | 2.67±0.71 | 2.82±0.70 |  | 1.17±0.33 | 1.18± 0.31 |
| Hansen PS [17] | 119 | 345 |  | 1.04±0.23 | 1.00±0.26 |  | - | - |  | 5.68±0.98 | 5.49±1.02 |  | - | - |  | - | - |
| Ukkola O [20] | 88 | 180 |  | - | - |  | 3.35±3.00 | 3.05±2.95 |  | 5.82±1.69 | 5.81±1.65 |  | 3.32±1.41 | 3.49±1.39 |  | 1.03±0.28 | 1.01±0.34 |
| Bohn M1 [11] | 35 | 153 |  | 1.44±0.30 | 1.61±0.37 |  | 1.76±0.95 | 2.06±1.40 |  | 6.29±0.96 | 6.92±1.27 |  | 4.58±0.84 | 5.20±1.16 |  | 0.88±0.29 | 0.85±0.23 |
| Bohn M2 [11] | 64 | 240 |  | 1.07±0.29 | 1.19±0.39 |  | 1.25±0.75 | 1.45±0.91 |  | 5.81±1.01 | 6.26±1.38 |  | 4.02±0.90 | 4.42±1.33 |  | 1.22±0.31 | 1.18±0.34 |
| Bohn M3 [11] | 10 | 40 |  | 1.42±0.52 | 1.70±0.41 |  | 1.52±1.21 | 1.79±0.89 |  | 7.39±1.68 | 7.58±1.42 |  | 5.46±1.77 | 5.66±1.28 |  | 1.16±0.43 | 1.03±0.27 |
| Bohn M4 [11] | 49 | 268 |  | 0.93±0.31 | 1.07±0.35 |  | 0.86±0.43 | 0.95±0.50 |  | 5.43±1.00 | 6.04±1.33 |  | 3.65±0.94 | 4.07±1.23 |  | 1.47±0.32 | 1.45±0.38 |
| Hansen PS1 [61] | 56 | 201 |  | - | - |  | - | - |  | 6.76±0.79 | 6.96±0.86 |  | - | - |  | - | - |
| Hansen PS2 [61] | 20 | 38 |  | - | - |  | - | - |  | 6.95±0.96 | 7.12±0.89 |  | - | - |  | - | - |
| Gajra B [60] | 155 | 32 |  | - | - |  | 1.33±0.79 | 1.60±0.89 |  | 4.09±1.45 | 4.36±1.93 |  | 2.82±1.29 | 3.09±1.89 |  | - | - |
| Pan JP1 [53] | 144 | 4 |  | 0.86±0.22 | 0.88±0.16 |  | 1.72±1.16 | 1.77±0.20 |  | 4.92±1.16 | 5.34±0.91 |  | 3.76±1.16 | 4.12±1.01 |  | 0.91±0.33 | 0.83±0.08 |
| Pan JP2 [53] | 151 | 2 |  | 0.80±0.13 | 0.98±0.01 |  | 1.31±0.61 | 2.71±0.91 |  | 4.90±0.96 | 5.96±2.43 |  | 3.44±1.01 | 5.15±2.02 |  | 0.98±0.36 | 0.70±0.39 |
| Ye P1 [36] | 85 | 103 |  | 0.83±0.18 | 0.79±0.11 |  | 1.56±0.34 | 1.81±0.30 |  | 5.62±2.02 | 5.45±1.07 |  | 3.57±0.99 | 3.31±0.85 |  | 0.96±0.23 | 0.85±0.20 |
| Ye P2 [36] | 95 | 100 |  | 0.69±0.13 | 0.69±0.13 |  | 1.16±0.48 | 1.33±0.67 |  | 4.33±1.03 | 4.37±0.87 |  | 2.65±0.88 | 2.68±1.29 |  | 1.24±0.16 | 1.22±0.26 |
| Pajukanta PE [38] | 34 | 53 |  | 1.01±0.24 | 1.04±0.20 |  | 1.40±0.60 | 1.40±0.68 |  | 5.54±0.86 | 5.51±0.81 |  | 3.29±0.76 | 3.37±0.66 |  | - | - |
| Guo Y [73] | 47 | 8 |  | - | - |  | 1.69±0.85 | 1.65 ±0.39 |  | 5.12±1.39 | 6.20±0.69 |  | 3.57±0.51 | 3.78±0.62 |  | 1.13±0.32 | 0.84±0.10 |
| Feng JS1 [72] | 117 | 11 |  | - | - |  | 0.99±0.34 | 1. 01±0. 27 |  | 4. 99±0. 85 | 4. 70±0. 67 |  | - | - |  | - | - |
| Feng JS2 [72] | 100 | 8 |  | - | - |  | 1. 42±0. 60 | 1. 55±1. 24 |  | 7. 16±0. 64 | 7. 16±0. 74 |  | - | - |  | - | - |
| Feng JS3 [72] | 49 | 3 |  | - | - |  | 2. 73±1. 35 | 2. 66±0. 67 |  | 6. 44±1. 16 | 5. 60±1. 54 |  | - | - |  | - | - |
| Zhao RZ [70] | 93 | 24 |  | 0.67±0.09 | 0.79±0.17 |  | 1.39±0.78 | 1.83±0.90 |  | 4.41±0.67 | 5.49±0.93 |  | 2.53±1.11 | 3.64±1.21 |  | 1.61±0.34 | 1.52±0.53 |
| Corbo RM1 [27] | 19 | 26 |  | - | - |  | - | - |  | 4.51±1.08 | 5.20±1.04 |  | - | - |  | - | - |
| Corbo RM2 [27] | 39 | 79 |  | - | - |  | - | - |  | 4.94±0.98 | 5.17±1.32 |  | - | - |  | - | - |
| Hubacek JA1 [54] | 15 | 67 |  | 1.14±0.37 | 1.04±0.39 |  | 1.00±0.51 | 0.99±0.41 |  | 5.60±0.73 | 5.47±0.66 |  | 4.11±0.76 | 3.99±0.64 |  | 1.43±0.28 | 1.45±0.35 |
| Hubacek JA2 [54] | 22 | 62 |  | 0.73±0.29 | 0.85±0.37 |  | 0.99±0.57 | 0.97±0.62 |  | 3.38±0.50 | 3.55±0.57 |  | 2.11±0.48 | 2.31±0.56 |  | 1.25±0.24 | 1.24±0.29 |
| Stepanov VA1 [63] | 25 | 67 |  | - | - |  | 1.42±0.40 | 1.31±0.40 |  | 6.06±1.07 | 6.15±1.08 |  | - | - |  | - | - |
| Stepanov VA2 [63] | 31 | 87 |  | - | - |  | 1.57±1.11 | 1.25±1.11 |  | 5.63±2.23 | 6.17±2.23 |  | - | - |  | 1.24±0.30 | 1.37±0.30 |
| Yao J1[71] | 25 | 67 |  | 1.09±0.23 | 1.12±0. 24 |  | 2.10±1.0 | 2.42±1.21 |  | 5.20±1.61 | 5.20±1.17 |  | 2.83±1.46 | 2.75±0.82 |  | 1.27±0.36 | 1.17±0.39 |
| Yao J2[71] | 31 | 87 |  | 0.96±0.21 | 1.02±0.3 |  | 1.31±0.35 | 1.82±0.93 |  | 4.4±0.97 | 5.0±1.42 |  | 2.5±0.95 | 2.50±1.12 |  | 1.35±0.4 | 1.44±0.48 |
| Corbo RM1 [37] | 44 | 97 |  | - | - |  | - | - |  | - | - |  | - | - |  | 1.02±0.22 | 0.96±0.29 |
| Corbo RM2 [37] | 39 | 69 |  | - | - |  | - | - |  | - | - |  | - | - |  | 1.23±0.29 | 1.20±0.28 |
| Korhonen T [18] | 14 | 35 |  | 1.11±0.19 | 1.42±0.28 |  | 1.98±1.06 | 2.14±1.11 |  | 7.03±0.82 | 8.08±1.04 |  | - | - |  | - | - |
| Choong ML [64] | 312 | 62 |  | 1.11±0.40 | 1.24±0.50 |  | - | - |  | 5.66±1.11 | 5.85±0.84 |  | 4.05±1.00 | 4.20±0.73 |  | 0.99±0.31 | 0.91±0.28 |
| Wang LY1 [8] | 134 | 16 |  | 0.78±0.17 | 0.89±0.29 |  | 1.40±0.60 | 1.60±10.00 |  | 5.20±1.30 | 5.50±1.00 |  | 3.20±1.20 | 3.20±0.90 |  | 1.80±0.60 | 1.80±0.50 |
| Wang LY2 [8] | 211 | 16 |  | 0.88±0.30 | 0.91±0.28 |  | 1.20±0.70 | 1.20±0.60 |  | 4.50±1.40 | 5.20±0.60 |  | 3.10±1.30 | 3.20±0.60 |  | 1.30±0.40 | 1.40±0.30 |
| Guzman EC1 [25] | 13 | 28 |  | - | - |  | - | - |  | 6.73±0.78 | 6.83±1.15 |  | 4.71±0.92 | 4.86±1.10 |  | - | - |
| Guzman EC2 [25] | 24 | 39 |  | - | - |  | - | - |  | 8.51±1.72 | 7.07±1.11 |  | 6.31±1.72 | 4.95±1.08 |  | - | - |
| Han T1 [12] | 150 | 40 |  | 0.93±0.22 | 0.99±0.13 |  | 1.35±0.99 | 1.38±1.28 |  | 4.54±0.89 | 4.84±0.74 |  | 2.49±0.69 | 2.74±0.58 |  | 1.32±0.33 | 1.33±0.30 |
| Han T2 [12] | 406 | 35 |  | 1.00±0.24 | 1.03±0.24 |  | 1.21±0.85 | 1.28±1.35 |  | 4.77±0.98 | 4.91±0.97 |  | 2.63±0.72 | 2.74±0.74 |  | 1.36±0.33 | 1.38±0.39 |
| Lopez-Miranda J [28] | 21 | 51 |  | 0.60±0.13 | 0.67±0.16 |  | 0.69±0.25 | 0.85±0.50 |  | 3.80±0.66 | 4.19±0.65 |  | 2.19±0.54 | 2.50±0.65 |  | 1.29±0.31 | 1.29±0.35 |
| Wei JB1 [55] | 85 | 25 |  | 0.73±0.02 | 0.75±0.02 |  | 1.20±0. 95 | 1. 41±1. 02 |  | 3. 60±0.94 | 4. 45±1.08 |  | 1. 98±0. 96 | 2. 64±0. 86 |  | 0. 94±0. 12 | 0. 97±0. 04 |
| Wei JB1 [55] | 56 | 6 |  | 0.59±0.02 | 0.59±0.02 |  | 1. 18±0. 75 | 1. 16±0. 78 |  | 4. 09±1.13 | 4. 15±1. 05 |  | 2. 37±0. 23 | 2. 42±0. 87 |  | 1. 19±0. 24 | 1. 21±0. 38 |
| Zhu WL1 [56] | 129 | 22 |  | 0.69±0.15 | 0.68±0.15 |  | 0.87±0.43 | 0.87±0.33 |  | 4.17±0.68 | 4.01±0.69 |  | 2.17±0.56 | 2.15±0.54 |  | 1.48±0.28 | 1.40±0.31 |
| Zhu WL1 [56] | 138 | 19 |  | 0.71±0.14 | 0.69±0.23 |  | 0.86±0.33 | 0.91±0.36 |  | 4.21±0.72 | 4.18±0.94 |  | 2.25±0.60 | 2.18±0.92 |  | 1.45±0.29 | 1.46±0.27 |
| Avakian SD1 [65] | 9 | 53 |  | - | - |  | 1.56±0.58 | 1.58±0.51 |  | 5.55±1.36 | 5.55±1.25 |  | 3.56±0.93 | 3.57±1.01 |  | 1.16±0.58 | 1.16±0.53 |
| Avakian SD2 [65] | 19 | 43 |  | - | - |  | 1.55±0.54 | 1.59±0.46 |  | 5.55±1.36 | 5.55±1.29 |  | 3.51±0.87 | 3.50±1.07 |  | 1.25±0.48 | 1.24±0.48 |
| Baroni MG [9] | 35 | 67 |  | 1.00±0.30 | 1.12±0. 22 |  | 2.37±0.64 | 2.39±0.76 |  | 4.94±0.88 | 5.33±0.91 |  | 3.57±0.91 | 3.85±0.93 |  | 0.75±0.25 | 0.67±0.05 |
| Li S [13] | 218 | 26 |  | 0.77±0.08 | 0.84±0.09 |  | 0.93±0.26 | 1.01±0.27 |  | 4.01±0.70 | 4.64±0.88 |  | 2.95±0.70 | 3.70 ±0.69 |  | 1.00±0.26 | 0.98±0.29 |
| Gong LG1 [80] | 85 | 29 |  | 0.91±0.63 | 1.18±0 .41 |  | 3.35±1.60 | 4 .15±1.36 |  | 4.01±0.98 | 5 .11±1.05 |  | 2.01±0.95 | 2 .95±0.91 |  | 0.95±0.12 | 0 .97±0 .12 |
| Gong LG1 [80] | 138 | 12 |  | 0 .79±0 .54 | 0 .72±0 .49 |  | 1 .14±0.75 | 1.17±0 .79 |  | 4 .03±1 .02 | 4 .29±1.07 |  | 2 .13±0.74 | 2 .18±0.89 |  | 1.07±0 .17 | 1.15±0 .29 |
| Tan YF1 [77] | 84 | 22 |  | 0 .91±0.28 | 0 .93±0.27 |  | 1 .35±1.02 | 1.54±1 .03 |  | 4 .34±1 .17 | 4 .63±1.21 |  | 2 .65±1.01 | 2 .85±0 .88 |  | 1 .04±0 .29 | 1 .05±0 .35 |
| Tan YF2 [77] | 94 | 11 |  | 0 .85±0.31 | 0 .72±0.21 |  | 1 .29±0.99 | 1.01±0 .98 |  | 4 .23±1 .32 | 4 .07±1.08 |  | 2 .29±0 .87 | 2 .19±0 .85 |  | 1 .13±0 .31 | 1 .38±0 .27 |
| Gong WD1 [79] | 84 | 22 |  | 0. 91±0. 28 | 0. 93±0. 27 |  | 1. 35±1. 02 | 1. 54±1. 03 |  | 4. 34±1. 17 | 4. 63±1. 21 |  | 2. 65±1. 01 | 2. 85±0. 88 |  | 1. 04±0. 29 | 1. 05±0. 35 |
| Gong WD2 [79] | 94 | 11 |  | 0. 85±0. 31 | 0. 72±0. 21 |  | 1. 29±0. 99 | 1. 01±0. 98 |  | 4. 23±1. 32 | 4. 07±1. 08 |  | 2. 29±0. 87 | 2. 19±0. 85 |  | 1. 13±0. 31 | 1. 38±0. 27 |
| Duman BS1 [66] | 47 | 103 |  | - | - |  | 1.84±1.24 | 1.52±0.82 |  | 5.06±1.64 | 5.17±1.42 |  | 3.24±1.33 | 3.50±1.19 |  | 1.11±0.66 | 1.13±0.75 |
| Duman BS2 [66] | 46 | 54 |  | - | - |  | 1.37±0.97 | 1.19±0.42 |  | 5.07±1.43 | 4.72±0.76 |  | 3.55±1.33 | 3.07±1.03 |  | 1.23±0.26 | 1.19±0.21 |
| Duman BS1 [67] | 48 | 60 |  | 1.17±0.28 | 1.10±0.58 |  | 1.55±7.00 | 2.37±12.96 |  | 4.50±12.47 | 5.00±13.09 |  | - | - |  | - | - |
| Duman BS2 [67] | 47 | 47 |  | 1.05±1.71 | 1.22±1.60 |  | 1.64±6.10 | 1.86±5.33 |  | 5.17±7.82 | 5.79±8.40 |  | - | - |  | - | - |
| Nieminen T [14] | 755 | 1461 |  | 1.04±0.26 | 1.08±0.27 |  | 1.30±0.78 | 1.36±0.89 |  | 5.11±0.95 | 5.19±1.00 |  | 3.22±0.85 | 3.30±0.86 |  | 1.31±0.33 | 1.28±0.32 |
| Hu P [75] | 116 | 10 |  | 0 .71±0 .08 | 1 .20±0.48 |  | 0 .88±0.44 | 3 .00±1.63 |  | 4 .02±0.54 | 9 .07±5 .22 |  | 2 .05±0 .36 | 5 .65±2 .94 |  | 1 .34±0.37 | 1 .79±1 .10 |
| Hu P [78] | 132 | 18 |  | 1.28±0.38 | 1.76±0.54 |  | 2.77±1.74 | 3.76±3.44 |  | 7.72±2.81 | 12.12±3.10 |  | 5.35±2.54 | 10.64±3.05 |  | 1.75±0.70 | 2.04±0.99 |
| Hu ZY1 [30] | 102 | 28 |  | 0.76±0.76 | 0.78±0.35 |  | 1.41±0.43 | 1.37±0.28 |  | 4.29±0.35 | 4.68±0.82 |  | 2.35±0.53 | 2.74±0.88 |  | 1.35±0.20 | 1.11±0.43 |
| Hu ZY2 [30] | 93 | 7 |  | 0.79±0.23 | 0.77±0.11 |  | 1.19±0.16 | 1.18±0.10 |  | 4.12±0.34 | 4.57±0.33 |  | 2.17±0.64 | 2.27±0.63 |  | 1.46±0.06 | 1.38±0.15 |
| Hu P [15] | 195 | 5 |  | 0.70±0.10 | 1.20±0.50 |  | 0.90±0.40 | 1.10±0.40 |  | 4.00±0.50 | 9.10±1.20 |  | 2.10±0.40 | 3.40±0.90 |  | 1.30±0.40 | 1.80±1.10 |
| Qian J [74] | 84 | 7 |  | - | - |  | - | - |  | 6 .21±0 .78 | 6 .68±1 .38 |  | 3.71±0 .78 | 3.56±1.07 |  | 1.22±0.30 | 1.21±0.39 |
| Park MH [22] | 6799 | 807 |  | - | - |  | 1.76±1.08 | 1.80±1.12 |  | - | - |  | 2.97±0.81 | 3.06±0.81 |  | - | - |
| Ma ZZ1 [76] | 198 | 52 |  | 0.97±0.37 | 0.98±0.37 |  | 2.35±0.87 | 2.51±0.83 |  | 5.25±0.92 | 5.78±0.95 |  | 3.35±0.72 | 3.53±0.80 |  | 2.33±0.37 | 2.28±0.35 |
| Ma ZZ2 [76] | 222 | 28 |  | 0.86±0.32 | 0.93±0.35 |  | 0.96±0.28 | 1.01±0.30 |  | 4.08±0.56 | 4.14±0.60 |  | 2.05±0.47 | 2.11±0.51 |  | 2.51±0.38 | 2.44±0.35 |
| Gallegos-Arreola MP1 [4] | 57 | 57 |  | - | - |  | 1.92±0.88 | 2.22±1.12 |  | 4.86±1.21 | 4.67±0.93 |  | 2.95±1.14 | 2.51±0.93 |  | 0.94±0.24 | 0.97±0.34 |
| Gallegos-Arreola MP2 [4] | 59 | 73 |  | - | - |  | 1.23±0.67 | 1.27±0.61 |  | 4.61±1.18 | 4.81±1.00 |  | 2.92±1.01 | 2.97±0.98 |  | 1.16±0.36 | 1.30±0.66 |
| Tsunoda K1 [21] | 41 | 7 |  | - | - |  | 0.98±0.47 | 1.31±0.37 |  | 3.73±0.77 | 3.70±0.65 |  | 2.04±0.65 | 2.03±0.46 |  | 1.25±0.29 | 1.06±0.31 |
| Tsunoda K2 [21] | 48 | 6 |  | - | - |  | 0.91±0.45 | 0.75±0.30 |  | 3.90±0.84 | 4.05±0.50 |  | 2.07±0.70 | 2.30±0.47 |  | 1.42±0.28 | 1.41±0.33 |
| Srivastava N1 [68] | 79 | 53 |  | - | - |  | 2.09±0.11 | 2.22±0.03 |  | 6.79±0.17 | 6.79±0.21 |  | 4.53±0.15 | 4.52±0.17 |  | 0.80±0.06 | 0.79±0.04 |
| Srivastava N2 [68] | 70 | 62 |  | - | - |  | 1.18±0.07 | 1.13±0.07 |  | 4.40±0.25 | 4.36±0.17 |  | 2.70±0.17 | 2.75±0.16 |  | 1.12±0.15 | 1.11±0.14 |
| Al-Bustan SA [57] | 343 | 323 |  | - | - |  | 1.06±0.93 | 1.06±0.82 |  | 4.70±0.93 | 4.70±0.99 |  | 3.13±0.91 | 3.11±0.80 |  | 1.12±0.36 | 1.13 ± 0.31 |
| Lazzaretti RK [26] | 122 | 285 |  | - | - |  | 2.11±1.42 | 2.24±2.16 |  | 4.73±1.09 | 5.09±1.23 |  | 2.46±0.91 | 2.79±0.88 |  | 1.34±0.39 | 1.30±0.35 |
| Berkinbayev S [16] | 156 | 85 |  | 0.98±0.28 | 1.02±0.19 |  | 2.17±0.60 | 2.24±0.52 |  | 5.36±0.98 | 5.49±0.82 |  | 3.93±0.79 | 4.04±0.67 |  | 1.00±0.32 | 1.01±0.34 |
| Liu YL [31] | 323 | 28 |  | 0.88±0.36 | 0.93±0.34 |  | 1.39±0.79 | 1.50±0.78 |  | 5.32±1.04 | 5.26±0.95 |  | 3.12±0.93 | 3.45±0.93 |  | 1.54±0.34 | 1.43±0.29 |
| Starcevic JN [69] | 158 | 437 |  | 0.90±0.22 | 0.91±0.21 |  | - | - |  | 4.58±1.11 | 4.74±1.21 |  | 2.58±0.88 | 2.65±0.95 |  | - | - |
| Kulminski AM1 [29] | 2378 | 7214 |  | - | - |  | - | - |  | 5.43±1.03 | 5.60±1.06 |  | - | - |  | 1.32±0.43 | 1.30±0.43 |
| Kulminski AM2 [29] | 2232 | 6243 |  | - | - |  | - | - |  | 4.99±1.05 | 5.14±1.06 |  | - | - |  | 1.37±0.39 | 1.35±0.40 |
| Kulminski AM3 [29] | 729 | 1956 |  | - | - |  | - | - |  | 5.01±0.94 | 5.08±0.91 |  | - | - |  | 1.37±0.41 | 1.35±0.41 |
| Kulminski AM4 [29] | 1176 | 3244 |  | - | - |  | - | - |  | 5.44±1.00 | 5.48±1.02 |  | - | - |  | 1.37±0.41 | 1.39±0.41 |

**Table S3:** Plasma APOB and lipid levels by the rs17240441 genotypes of individual studies included in the meta-analysis.

| **First author, reference** | **Genotype** | |  | **APOB, mg/dL** | |  | **TG, mmol/L** | |  | **TC, mmol/L** | |  | **LDL-C, mmol/L** | |  | **HDL-C, mmol/L** | |
| --- | --- | --- | --- | --- | --- | --- | --- | --- | --- | --- | --- | --- | --- | --- | --- | --- | --- |
|  | II | ID+DD |  | II | ID+DD |  | II | ID+DD |  | II | ID+DD |  | II | ID+DD |  | II | ID+DD |
| Hegele RA1 [47] | 46 | 38 |  | 0.98±0.25 | 1.04±0.25 |  | 1.93±1.01 | 1.87±1.02 |  | 5.73±1.11 | 5.75±1.09 |  | 3.67±0.95 | 3.73±0.95 |  | 0.98±0.25 | 0.94±0.21 |
| Hegele RA2 [47] | 62 | 22 |  | 0.97±0.22 | 0.95±0.28 |  | 1.72±1.02 | 1.40±0.92 |  | 5.90±1.12 | 5.90±1.12 |  | 3.69±0.89 | 3.57±0.98 |  | 1.15±0.34 | 1.12±0.28 |
| Xu CF [43] | 47 | 49 |  | 1.20±0.29 | 1.14±0. 25 |  | 1.30±0.70 | 0.98±0.40 |  | 6.30±1.10 | 6.14±1.20 |  | 4.60±1.10 | 4.52±1.08 |  | 1.40±0.30 | 1.42±0.31 |
| Renges HH [34] | 71 | 36 |  | - | - |  | - | - |  | 5.73±1.01 | 6.28±1.35 |  | - | - |  | 1.34 ±0.42 | 1.21±0.38 |
| Saha N [44] | 166 | 97 |  | 0.76±0.57 | 0.83±0.66 |  | 1.51±1.85 | 1.43±1.70 |  | 5.65±2.60 | 6.45±2.97 |  | 3.65±2.47 | 4.46±2.67 |  | 1.31±0.70 | 1.32±0.69 |
| Gaffney D1 [59] | 139 | 221 |  | - | -- |  | 1.78±0.87 | 1.88±0.76 |  | - | - |  | 4.94±0.55 | 4.99±0.62 |  | - | - |
| Gaffney D2 [59] | 80 | 108 |  | - | - |  | 1.17±0.56 | 1.22±0.58 |  | - | - |  | 3.46±0.96 | 3.57±0.99 |  | - | - |
| Saha N [52] | 129 | 33 |  | 2.53±1.53 | 2.49±2.27 |  | 1.57±2.08 | 2.59±3.32 |  | 5.33±2.23 | 6.01±3.40 |  | 3.63±2.09 | 3.88±3.24 |  | 0.88±0.88 | 1.02±0.73 |
| Bohn M1 [45] | 88 | 150 |  | 1.53±0.38 | 1.61±0.38 |  | 1.97±1.32 | 1.89±1.23 |  | 6.77±1.24 | 7.04±1.35 |  | 4.98±1.16 | 5.32±1.23 |  | 0.89±0.30 | 0.89±0.25 |
| Bohn M2 [45] | 215 | 332 |  | 1.09±0.36 | 1.13±0.36 |  | 1.14±0.70 | 1.16±0.71 |  | 5.86±1.33 | 6.16±1.34 |  | 4.06±1.24 | 4.34±1.27 |  | 1.34±0.39 | 1.31±0.37 |
| Hansen PS1 [61] | 100 | 158 |  | - | - |  | - | - |  | 6.85±0.81 | 6.96±0.87 |  | - | - |  | - | - |
| Hansen PS2 [61] | 29 | 30 |  | - | - |  | - | - |  | 6.92±0.97 | 7.20±0.85 |  | - | - |  | - | - |
| Ye P1 [36] | 54 | 49 |  | 0.80±0.20 | 0.84±0.31 |  | 1.46±0.84 | 1.77±0.78 |  | 5.65±1.17 | 5.46±0.98 |  | 3.58±0.90 | 3.32±0.97 |  | 0.96±0.21 | 0.83±0.22 |
| Ye P2 [36] | 59 | 41 |  | 0.70±0.12 | 0.67±0.14 |  | 1.18±0.48 | 1.16±0.51 |  | 4.13±0.96 | 4.59±1.06 |  | 2.54±0.88 | 2.84±0.90 |  | 1.27±0.17 | 1.15±0.16 |
| Pajukanta PE [38] | 42 | 45 |  | 0.97±0.23 | 1.08±0.19 |  | 1.34±0.58 | 1.45±0.70 |  | 5.31±0.79 | 5.72±0.80 |  | 3.20±0.70 | 3.49±0.68 |  | - | - |
| Hong SH1 [41] | 92 | 72 |  | - | - |  | 1.44±0.61 | 1.75±0.92 |  | 4.56±0.73 | 4.75±0.80 |  | 3.04±0.64 | 3.04±0.75 |  | 0.89±0.20 | 0.93±0.23 |
| Hong SH2 [41] | 65 | 97 |  | - | - |  | 1.56±0.58 | 1.72±0.88 |  | 4.60±0.57 | 4.68±0.63 |  | 2.96±0.54 | 2.97±0.62 |  | 0.92±0.21 | 0.93±0.26 |
| Hong SH3 [41] | 37 | 34 |  | - | - |  | 1.35±0.62 | 1.93±1.07 |  | 5.07±1.56 | 4.62±0.90 |  | 2.89±0.83 | 3.28±0.60 |  | 0.89± 0.20 | 0.94±0.23 |
| Hong SH4 [41] | 20 | 34 |  | - | - |  | 1.31±0.88 | 1.44±0.61 |  | 1.96±0.40 | 5.15±0.85 |  | 3.50±1.45 | 2.99±0.70 |  | 0.95±0.22 | 0.98±0.28 |
| Glisic S1 [62] | 72 | 150 |  | - | - |  | 1.19±0.14 | 1.19±0.15 |  | 4.68±0.20 | 4.70±0.24 |  | 2.66±0.16 | 2.68±0.19 |  | 1.48±0.06 | 1.48±0.05 |
| Glisic S2 [62] | 53 | 179 |  | - | - |  | 1.03±0.12 | 1.04±0.15 |  | 4.89±0.19 | 4.90±0.23 |  | 2.79±0.12 | 2.80±0.14 |  | 1.63±0.08 | 1.62±0.07 |
| Gardemann A [39] | 1075 | 1184 |  | 1.26±0.35 | 1.29±0.34 |  | 1.73±1.08 | 1.74±0.99 |  | 5.35±1.09 | 5.48±1.12 |  | - | - |  | - | - |
| Lu XY [81] | 56 | 44 |  | 0.60±0.20 | 0.66±0.13 |  | 0.82±0.42 | 0.80±0.46 |  | 3.42±0.62 | 3.48±0.56 |  | 1.75±0.21 | 1.93±0.25 |  | 1.25±0.52 | 1.23±0.62 |
| Choong ML [40] | 357 | 179 |  | 1.20±0.43 | 1.33±0.48 |  | - | - |  | 5.80±1.15 | 6.17±1.44 |  | 4.08±1.05 | 4.46±1.33 |  | 0.99±0.33 | 0.99±0.30 |
| Guzmán EC [25] | 22 | 32 |  | - | - |  | - | - |  | 7.56 ±1.17 | 7.62±1.17 |  | 5.41±1.17 | 5.43±1.16 |  | - | - |
| Avakian SD1 [65] | 34 | 28 |  | - | - |  | 1.56±0.58 | 1.54±0.56 |  | 5.54±1.15 | 5.55±1.16 |  | 3.42±1.23 | 3.51±1.16 |  | 1.16±0.54 | 1.23±0.49 |
| Avakian SD2 [65] | 43 | 19 |  | - | - |  | 1.54±0.45 | 1.56±0.54 |  | 5.54±1.15 | 5.56±1.13 |  | 3.54±0.78 | 3.49±0.94 |  | 1.28±0.42 | 1.29±0.39 |
| Jemaa R [46] | 121 | 110 |  | 0.98±0.32 | 1.04±0.34 |  | 1.48±1.49 | 1.50±1.30 |  | 5.50±0.98 | 5.76±1.11 |  | 3.61±0.88 | 3.89±0.96 |  | 1.16±0.28 | 1.15±0.26 |
| Rebhi L [6] | 48 | 109 |  | 1.07±0.20 | 1.12±0.38 |  | 1.40±0.70 | 1.55±1.06 |  | 4.90±1.10 | 5.23±1.09 |  | 3.20±0.90 | 3.56±0.98 |  | 1.01±0.27 | 0.96±0.25 |
| Tsunoda K1 [21] | 39 | 9 |  | - | - |  | 0.99±0.49 | 1.17±0.29 |  | 3.74 ±0.70 | 3.84±0.89 |  | 1.98±0.62 | 2.25±0.63 |  | 1.26±0.29 | 1.04±0.27 |
| Tsunoda K2 [21] | 43 | 11 |  | - | - |  | 0.87±0.43 | 0.83±0.37 |  | 3.92±0.76 | 3.74±0.93 |  | 2.12±0.67 | 1.98±0.77 |  | 1.40±0.26 | 1.37±0.31 |
| Lazzaretti RK [26] | 193 | 215 |  | - | - |  | 2.20±1.89 | 2.18±2.03 |  | 4.89±1.09 | 5.07±1.30 |  | 2.56±0.80 | 2.81±0.96 |  | 1.34±0.39 | 1.31±0.36 |
| Al-Bustan SA [57] | 395 | 271 |  | - | - |  | 1.11±0.79 | 0.98±0.77 |  | 4.71±0.99 | 4.69±0.91 |  | 3.13±0.77 | 3.11±0.87 |  | 1.11±0.19 | 1.15±0.30 |
| Vimaleswaran KS [42] | 25 | 122 |  | - | - |  | 2.25±0.70 | 1.82±0.78 |  | 6.43±1.05 | 6.05±0.93 |  | 4.32±0.95 | 4.03±0.87 |  | 1.04±0.20 | 1.20±0.28 |

**Figure S1:** Galbraith plot for the association analysis between the *APOB* rs693 polymorphism and APOB.

**Figure S2:** Galbraith plot for the association analysis between the *APOB* rs693 polymorphism and TG.

**Figure S3:** Galbraith plot for the association analysis between the *APOB* rs693 polymorphism and TC.

**Figure S4:** Galbraith plot for the association analysis between the *APOB* rs693 polymorphism and LDL-C.

**Figure S5:** Galbraith plot for the association analysis between the *APOB* rs693 polymorphism and HDL-C.

**Figure S6:** Galbraith plot for the association analysis between the *APOB* rs17240441 polymorphism and TG.

**Figure S7:** Galbraith plot for the association analysis between the *APOB* rs17240441 polymorphism and TC.

**Figure S8:** Galbraith plot for the association analysis between the *APOB* rs17240441 polymorphism and LDL-C.

**Figure S9:** Galbraith plot for the association analysis between the *APOB* rs17240441 polymorphism and HDL-C.

**Figure S10:** Begg’s funnel plot for the association analysis between the *APOB* rs693 polymorphism and APOB (*Z* = 1.22, *P* = 0.22).

**Figure S11:** Begg’s funnel plot for the association analysis between the *APOB* rs693 polymorphism and TG (*Z* = 0.54, *P* = 0.59).

**Figure S12:** Begg’s funnel plot for the association analysis between the *APOB* rs693 polymorphism and TC (*Z* = 1.97, *P* = 0.05).

**Figure S13:** Begg’s funnel plot for the association analysis between the *APOB* rs693 polymorphism and LDL-C (*Z* = 0.88, *P* = 0.38).

**Figure S14:** Begg’s funnel plot for the association analysis between the *APOB* rs693 polymorphism and HDL-C (*Z* = 0.67, *P* = 0.50).

**Figure S15:** Begg’s funnel plot for the association analysis between the *APOB* rs17240441 polymorphism and APOB (*Z* = 0.00, *P* = 1.00).

**Figure S16:** Begg’s funnel plot for the association analysis between the *APOB* rs17240441 polymorphism and TG (*Z* = 0.39, *P* = 0.69).

**Figure S17:** Begg’s funnel plot for the association analysis between the *APOB* rs17240441 polymorphism and TC (Z = 0.47, *P* = 0.64).

**Figure S18:** Begg’s funnel plot for the association analysis between the *APOB* rs17240441 polymorphism and LDL-C (*Z* = 1.53, *P* = 0.13).

**Figure S19:** Begg’s funnel plot for the association analysis between the *APOB* rs17240441 polymorphism and HDL-C (*Z* = 0.38, *P* = 0.71).

**Figure S20:** Cumulative analysis plot according to the publication years for the association analysis between the *APOB* rs693 polymorphism and APOB.

**Figure S21:** Cumulative analysis plot according to the publication years for the association analysis between the *APOB* rs693 polymorphism and TG.

**Figure S22:** Cumulative analysis plot according to the publication years for the association analysis between the *APOB* rs693 polymorphism and TC.

**Figure S23:** Cumulative analysis plot according to the publication years for the association analysis between the *APOB* rs693 polymorphism and LDL-C.

**Figure S24:** Cumulative analysis plot according to the publication years for the association analysis between the *APOB* rs693 polymorphism and HDL-C.

**Figure S25:** Cumulative analysis plot according to the publication years for the association analysis between the *APOB* rs17240441 polymorphism and APOB.

**Figure S26:** Cumulative analysis plot according to the publication years for the association analysis between the *APOB* rs17240441 polymorphism and TG.

**Figure S27:** Cumulative analysis plot according to the publication years for the association analysis between the *APOB* rs17240441 polymorphism and TC.

**Figure S28:** Cumulative analysis plot according to the publication years for the association analysis between the *APOB* rs17240441 polymorphism and LDL-C.

**Figure S29:** Cumulative analysis plot according to the publication years for the association analysis between the *APOB* rs17240441 polymorphism and HDL-C.
